# High‐Performance Phototransistor Based on a 2D Polybenzimidazole Polymer

**DOI:** 10.1002/adma.202505810

**Published:** 2025-06-01

**Authors:** Anupam Prasoon, Preetam Dacha, Heng Zhang, Elif Unsal, Mike Hambsch, Alexander Croy, Shuai Fu, Nguyen Ngan Nguyen, Kejun Liu, Haoyuan Qi, Sein Chung, Minyoung Jeong, Lei Gao, Ute Kaiser, Kilwon Cho, Hai I. Wang, Renhao Dong, Gianaurelio Cuniberti, Mischa Bonn, Stefan C. B. Mannsfeld, Xinliang Feng

**Affiliations:** ^1^ Center for Advancing Electronics Dresden (cfaed) and Faculty of Chemistry and Food Chemistry Technische Universität Dresden 01062 Dresden Germany; ^2^ Max Planck Institute for Microstructure Physics D‐06120 Halle Germany; ^3^ Center for Advancing Electronics Dresden (cfaed) Faculty of Electrical and Computer Engineering Technische Universität Dresden 01062 Dresden Germany; ^4^ Max Planck Institute for Polymer Research Ackermannweg 10 55128 Mainz Germany; ^5^ Institute for Materials Science and Max Bergmann Center of Biomaterials Technische Universität Dresden 01062 Dresden Germany; ^6^ Institute of Physical Chemistry Friedrich Schiller University Jena 07737 Jena Germany; ^7^ Central Facility for Electron Microscopy Electron Microscopy of Materials Science Ulm University 89081 Ulm Germany; ^8^ Department of Chemical Engineering Pohang University of Science and Technology Pohang 37673 Republic of Korea; ^9^ Nanophotonics Debye Institute for Nanomaterials Science Utrecht University Utrecht 3584 Netherlands; ^10^ Department of Chemistry The University of Hong Kong Hong Kong 999077 China; ^11^ Materials Innovation Institute for Life Sciences and Energy (MILES) HKU‐SIRI Shenzhen 518048 China; ^12^ Dresden Center for Computational Materials Science (DCMS) Technische Universität Dresden 01062 Dresden Germany

**Keywords:** 2D polymers, on‐water surface synthesis, photodetectors, photoresponsive 2D polymers, phototransistors

## Abstract

Photodetectors are fundamental components of modern optoelectronics, enabling the conversion of light into electrical signals. The development of high‐performance phototransistors necessitates materials with both high charge carrier mobility and robust photoresponse. However, achieving both in a single material poses challenges due to inherent trade‐offs. Herein, this study introduces a polybenzimidazole‐(1,3‐diazole)‐based 2D polymer (2DPBI), synthesized as few‐layer, crystalline films covering ≈28 cm^2^ on the water surface at room temperature, with large crystalline domain sizes ranging from 110 to 140 µm^2^. The 2DPBI incorporates a π‐conjugated photoresponsive porphyrin motif through a 1,3‐diazole linkage, exhibiting enhanced π‐electron delocalization, a narrow direct band gap of ≈1.18 eV, a small reduced electron–hole effective mass (m* = 0.171 *m*_0_), and a very high resonant absorption coefficient of up to 10^6^ cm^−1^. Terahertz spectroscopy reveals excellent short‐range charge carrier mobility of ≈240 cm^2^ V^−1^ s^−1^. Temperature‐dependent photoconductivity measurements and theoretical calculations confirm a band‐like charge transport mechanism. Leveraging these features, 2DPBI‐based phototransistors demonstrate an on/off ratio exceeding 10^8^, photosensitivity of 1.08 × 10^7^, response time of 1.1 ms, and detectivity of 2.0 × 10^13^ Jones, surpassing previously reported standalone few‐layer 2D materials and are on par with silicon photodetectors. The unique characteristics of 2DPBI make it a promising foundation for future optoelectronic devices.

## Graphical abstract

A crystalline 2D polybenzimidazole polymer (2DPBI), incorporating a porphyrin‐based π‐system linked via 1,3‐diazole units, is synthesized on the water surface over large areas. The 2DPBI thin‐film exhibits high charge carrier mobility (≈240 cm^2^ V^−1^ s^−1^, measured by terahertz spectroscopy), strong photoresponse, and band‐like transport, enabling phototransistor performance with a detectivity of 2.0 × 10^13^ Jones, highlighting its potential for high‐performance optoelectronic applications.

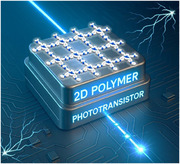

## 1 Introduction

The rapid advancement of optoelectronic devices has profoundly shaped 21st‐century technology.^[^ ^1^ ^]^ At the forefront of this revolution are phototransistors, which integrate light detection and signal amplification to power next‐generation applications such as digital imaging, optical communication, and logic gates.^[^ ^2^ ^]^ High‐performance phototransistors demand materials with high charge carrier mobility and robust photoresponsive characteristics, characterized by a high absorption coefficient and long free carrier lifetime.^[^ ^3^ ^]^ The adoption of 2D materials has led to substantial progress in optoelectronic devices, with phototransistors being a notable example of this advancement.^[^ ^4^ ^]^ Graphene and transition metal dichalcogenides (TMDs) offer remarkable mobilities, up to 10^5^ cm^2^ V^−1^ s^−1^ for graphene^[^ ^5^ ^]^ and 200 cm^2^ V^−1^ s^−1^ for monolayer MoS_2_.^[^ ^6^ ^]^ However, the photoresponsivity of graphene‐based photodetectors is mainly limited by the relatively weak and featureless light absorption of graphene, while the photogenerated electron‐hole pairs in TMDs are difficult to separate under weak electric fields due to their large exciton binding energy.^[^ ^7^ ^]^ Organic phototransistors (OPTs) present a complementary alternative with low dark currents (≈10^−10^–10^−12^ A). Yet, conventional semiconducting polymers typically suffer from low carrier mobility (<1 cm^2^V^−1^ s^−1^) due to long‐range structural and thermal disorders.^[^ ^3^, ^8^ ^]^ Recent advancements in high‐performance phototransistor devices have focused on creating heterostructures by assembling 2D materials like graphene and MoS_2_ or sensitizing 2D materials using organic molecules such as rubrene, fullerenes, and pentacene.^[^ ^2^, ^7^, ^9^ ^]^ These devices have demonstrated promising performance, with detectivity ranging from 10^6^ to 10^14^ Jones.^[^ ^2^, ^10^ ^]^ Despite these advancements, heterojunctions often lead to high surface charge traps, increased contact resistance, unintended interfacial doping, and hindering large‐scale fabrication.^[^ ^11^ ^]^ Addressing such challenges necessitates the development of novel semiconducting materials that offer solution processability, large‐area thin‐film synthesis, and simultaneously provide high optical absorbance and charge carrier mobility within a single material. The ongoing evolution of phototransistor technology continues to reflect the trajectory of Moore's Law, underscoring the urgent need for innovative materials and designs to meet the escalating demands of modern optoelectronic applications.^[^ ^12^ ^]^

Recently, 2D polymers (2DPs) and their layer‐stacked counterparts, 2D covalent organic frameworks (2D COFs), have emerged as promising candidates for optoelectronic applications.^[^ ^13^ ^]^ 2DPs are molecularly thin, possess extensive lateral dimensions, and form covalent frameworks with long‐range ordering in two distinct directions.^[^ ^14^ ^]^ Their versatility, highlighted by tunable bandgaps (broader energy level structures), enhanced light emission,^[^ ^15^ ^]^ and high quantum yields,^[^ ^16^ ^]^ positions them as a promising foundation for advanced optoelectronic devices.^[^ ^17^ ^]^ Despite the growing interest in 2DPs, these materials typically exhibit poor π‐electron delocalization in polymer backbones and show weak or moderate in‐plane dispersion in the energy band diagram.^[^ ^18^ ^]^ This leads to large bandgaps and suboptimal charge transport properties, posing significant challenges to the development of high‐performance optoelectronic devices based on 2DPs and 2D COFs.^[^ ^13^, ^19^ ^]^

This study introduces a polybenzimidazole (1,3‐diazole)‐based 2D polymer (2DPBI) that exhibits excellent charge carrier mobility and a high absorption coefficient. To enhance the degree of extended π‐conjugation and photoresponsive properties of 2DP, a conjugated π‐electron photoresponsive porphyrin motif is integrated into the backbone of the 2DP framework through a 1,3‐diazole linkage featuring a 5‐membered imidazole ring. This integration substantially promotes π‐electron delocalization. Consequently, the obtained 2DPBI possesses a narrow bandgap of ≈1.18 eV, a low reduced electron‐hole effective mass (m*) of 0.171 *m*_0_, and a high absorption coefficient of up to 10^6^ cm^−1^, comparable to commercial inorganic‐based semiconductors such as GaAs, Ge, and CdS.^[^ ^20^ ^]^ We synthesized few‐layer (thickness ≈2.4 nm, ≈5–6 layers), crystalline 2DPBI films with large crystalline domain sizes ranging from 110 to 140 µm^2^, covering an area of ≈28 cm^2^. This was achieved using a pH‐controlled irreversible 1,3‐diazole (imidazole) reaction between 5,10,15,20‐(tetra‐4‐carboxyphenyl) porphyrin (M1) and 1,2,4,5‐benzenetetramine (M2) through the stepwise sequential assembly of the monomers on the water surface at room temperature. Terahertz (THz) spectroscopy demonstrates an excellent charge carrier mobility of ≈240 cm^2^ V^−1^ s^−1^ at room temperature. The combination of temperature‐dependent photoconductivity measurements and theoretical calculations further shows that band‐like transport enables the outstanding charge transport properties. Extending our designed 2DPBI to optoelectronic applications, we successfully fabricated phototransistor devices, exhibiting an on/off ratio exceeding 10^8^, a photosensitivity of 1.08 × 10^7^, a photoresponsivity of 32 AW^−1^, a response time of 1.1 ms, and specific detectivity of 7.1 × 10^12^ Jones and 2.0 × 10^13^ Jones (based on noise current measurements and dark current measurements, respectively). These performance metrics surpass those of previously reported standalone few‐layer 2D materials (10^7^–10^11^ Jones),^[^ ^2^, ^10^ ^]^ compete with the highest‐performing solution‐processed semiconductor materials, typically with detectivity values in the range of 10^6^–10^11^ Jones,^[^ ^8^, ^21^ ^]^ and are competitive with silicon photodetectors (10^12^ Jones).^[^ ^22^ ^]^ These results establish 2DPBI as a promising candidate for next‐generation multifunctional optoelectronic devices.

## 2 Results and Discussion

### 2.1 Design Principle for a Conjugated and Photoresponsive 2D Polymer

**Figure 1:**
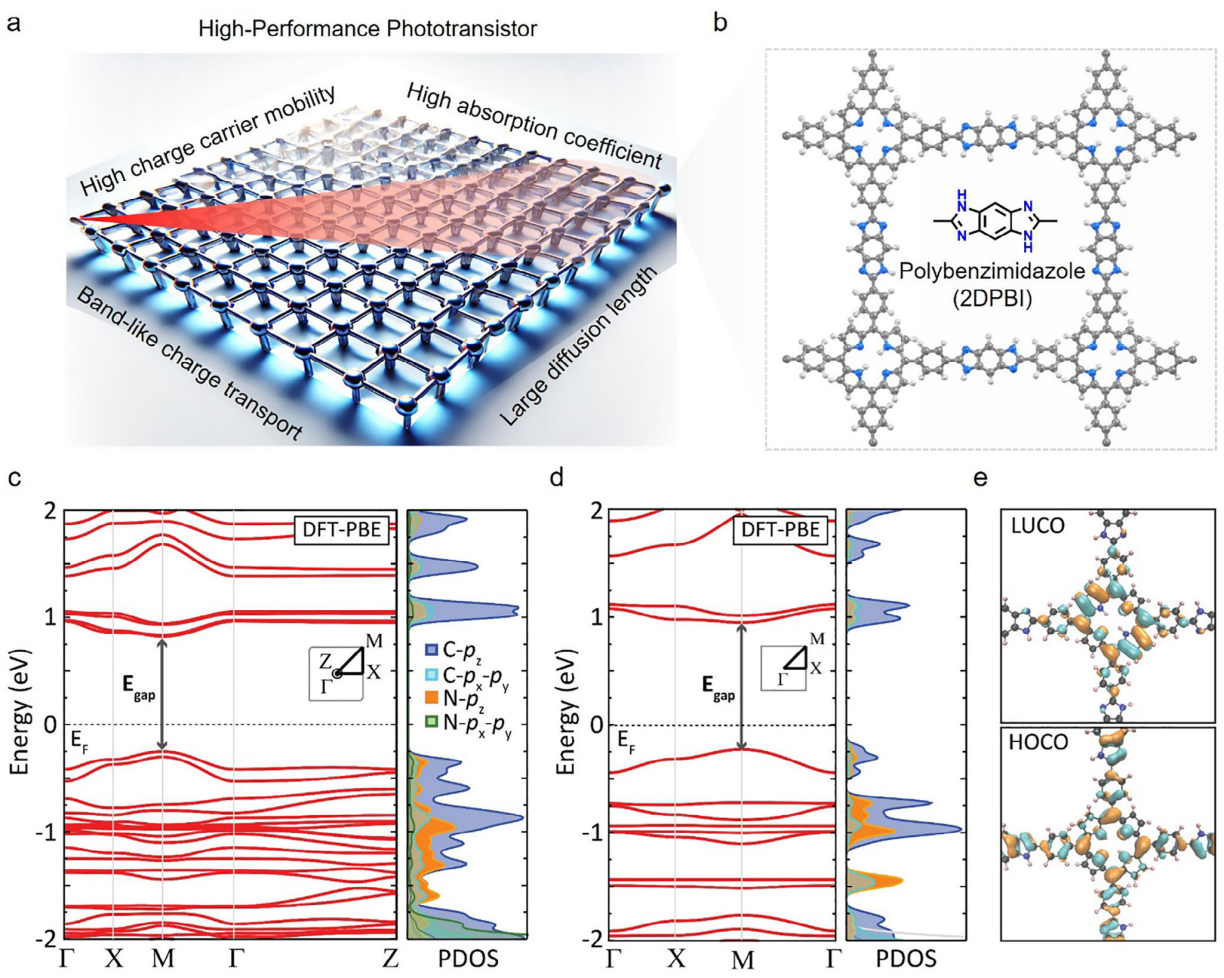
Structural design for a conjugated and photoresponsive 2D polymer. Schematic illustration of (a) the 2DP design and (b) porphyrin‐based 2DPs connected by 1,3‐diazole linkages. The calculated electronic band structures for (c) the layered bulk 2DPBI and (d) the monolayer 2DPBI. The electronic band gaps are 1.18 and 1.07 eV for the monolayer 2DPBI and its layered‐bulk counterpart, respectively. The partial density of states (PDOS) is shown in arbitrary units and the Fermi levels (E_F_) are set at zero. The Brillouin zones and the selected k‐paths are depicted in the insets. e) Visualizations of the lowest unoccupied crystal orbital (LUCO) and the highest occupied crystal orbital (HOCO) for the monolayer 2DPBI, with the positive and negative parts of the wavefunction represented in cyan and orange, respectively. The chosen isovalue is 0.02.

To endow 2DPs with enhanced photoresponsive properties through extensive π‐conjugation, porphyrin is selected as the structural backbone (**Figure** **1**a,b). Renowned for its conjugated π‐electron system, porphyrin facilitates effective charge transfer and robust light absorption, achieving absorption coefficients up to 10^5^ cm^−1^ in the Soret band (400–450 nm) and 10^4^ cm^−1^ in the Q‐bands (500–700 nm).^[^ ^23^ ^]^ Combining an intuitive chemistry design with theoretical computations, we explored various chemical linkages to extend the π‐conjugation and delocalization in the 2DP framework (Figures S1 and S2, Supporting Information). Specifically, the incorporation of a π‐conjugated 1,3‐diazole linkage featuring a 5‐membered imidazole ring within the 2DPBI structure results in enhanced delocalization of π‐electrons, significantly increasing the light absorption coefficient to 10^6^ cm^−1^ compared to the pre‐assembled M1 porphyrin monomer film (10^4^–10^5^ cm^−1^) (Figure S3, Supporting Information). The electronic band structure and density of states (DOS) obtained from theoretical calculations reveal a direct band gap at the high‐symmetry M point in 2DPBI, with significant contributions from carbon and nitrogen p‐orbitals. The partial density of states (PDOS) calculations suggest hybridization between the C‐*pz*, C‐*px‐py*, and N‐*pz* states within the conduction and valence bands, as depicted in Figure 1c. The calculated effective mass values for electrons and holes are 0.42 *m*_0_ and 0.29 *m*_0_, respectively (Figure 1c–e). The synergistic effects, including the reduced band gap, lower effective mass, extended π‐conjugation, and enhanced resonant absorption coefficient, make 2DPBI promising for optoelectronic applications.

### 2.2 On‐Water Surface Synthesis of 2DPBI

**Figure 2:**
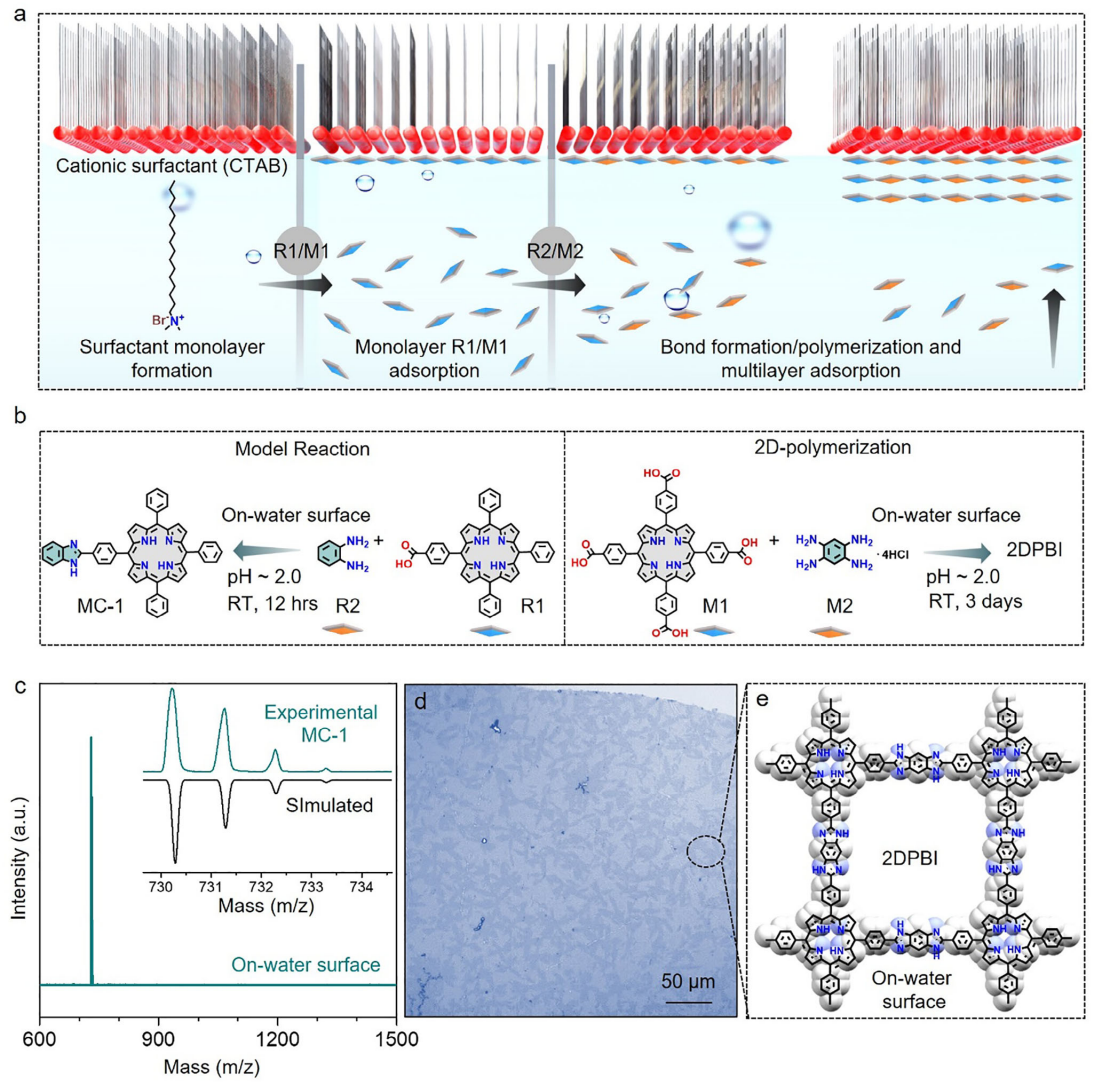
On‐water surface synthesis of 2DPBI. a,b) A schematic illustration depicting the (1,3‐diazole) imidazole model reaction and subsequent 2D polymerization process leading to the formation of 2DPBI on the water surface. c) MALDI‐TOF mass spectra of MC‐1 synthesized on the water surface, with an inset showing the HR‐MALDI‐TOF mass spectra of the MC‐1 model compound. d,e) Optical microscope image and molecular structure of 2DPBI synthesized on the water surface.

The synthesis of 2DPBI involves stepwise sequential assembly and reaction of monomers on the water surface under ambient conditions, facilitated by a charged surfactant monolayer (**Figure** **2**a).^[^ ^14^, ^24^ ^]^ The feasibility of the irreversible 1,3‐diazole (imidazole) reaction on the water surface was first evaluated using a model reaction between 4‐(10,15,20‐triphenylporphyrin‐5‐yl)benzoic acid (R1) and benzene‐1,2‐diamine (R2) under controlled pH conditions, as illustrated in Figure 2b. The model reaction on the water surface involved three steps: Step‐I, spreading of cetyltrimethylammonium bromide (CTAB), cationic surfactant on the water surface in a beaker of 6 cm diameter, resulting in the formation of a surfactant monolayer; Step‐II, after 10 min, an aqueous basic solution of R1 (pH ≈12.8) was injected into the water subphase, leading to the pre‐organization of the R1 monolayer beneath the surfactant monolayer, facilitated by the electrostatic interaction between R1 and the cationic head group of CTAB; Step‐III, after 1 h, an aqueous acidic solution of R2 (pH ≈2.0) was added to the water subphase, inducing its diffusion toward the pre‐organized monolayer of R1. The reaction was maintained at room temperature under ambient conditions for 12 h, culminating in the formation of a macroscopic shiny dark brownish‐colored film on the water surface, observable upon visual inspection. The resultant film was subsequently fished out from the water surface and analyzed using matrix‐assisted laser desorption/ionization–time‐of‐flight mass spectrometry (MALDI–TOF MS), revealing a single peak at an m/z value of 730.48 with an isotopic distribution corresponding to the final product 5‐(4‐(1H‐benzo[d]imidazol‐2‐yl)phenyl)‐10,15,20‐triphenylporphyrin (MC‐1) (Figure 2c). In contrast, when the same chemical reaction was performed in water under similar experimental conditions (pH, temperature, time, and concentration) without using a surfactant monolayer, only the reagent molecules were identified by mass spectrometry (Figure S4, Supporting Information). This control experiment suggests that the on‐water surface confinement of porphyrin molecules, reinforced by the surfactant monolayer, is imperative for enhanced chemical reactivity. pH‐ and time‐dependent studies further demonstrate that 1,3‐diazole (imidazole) cyclization proceeds selectively and efficiently under acidic conditions on the water surface (Figure S5, Supporting Information).

Following the experimental demonstration of the model reaction, we further explored 2D polymerization through an (A_4_+B_2_)‐type monomer for the 1,3‐diazole (imidazole) reaction between M1 and M2 to synthesize 2DPBI, under the same reaction conditions but extending the duration of step‐III to up to three days (Figure 2d,e).

### 2.3 Morphological and Structural Characterizations of 2DPBI Film

**Figure 3:**
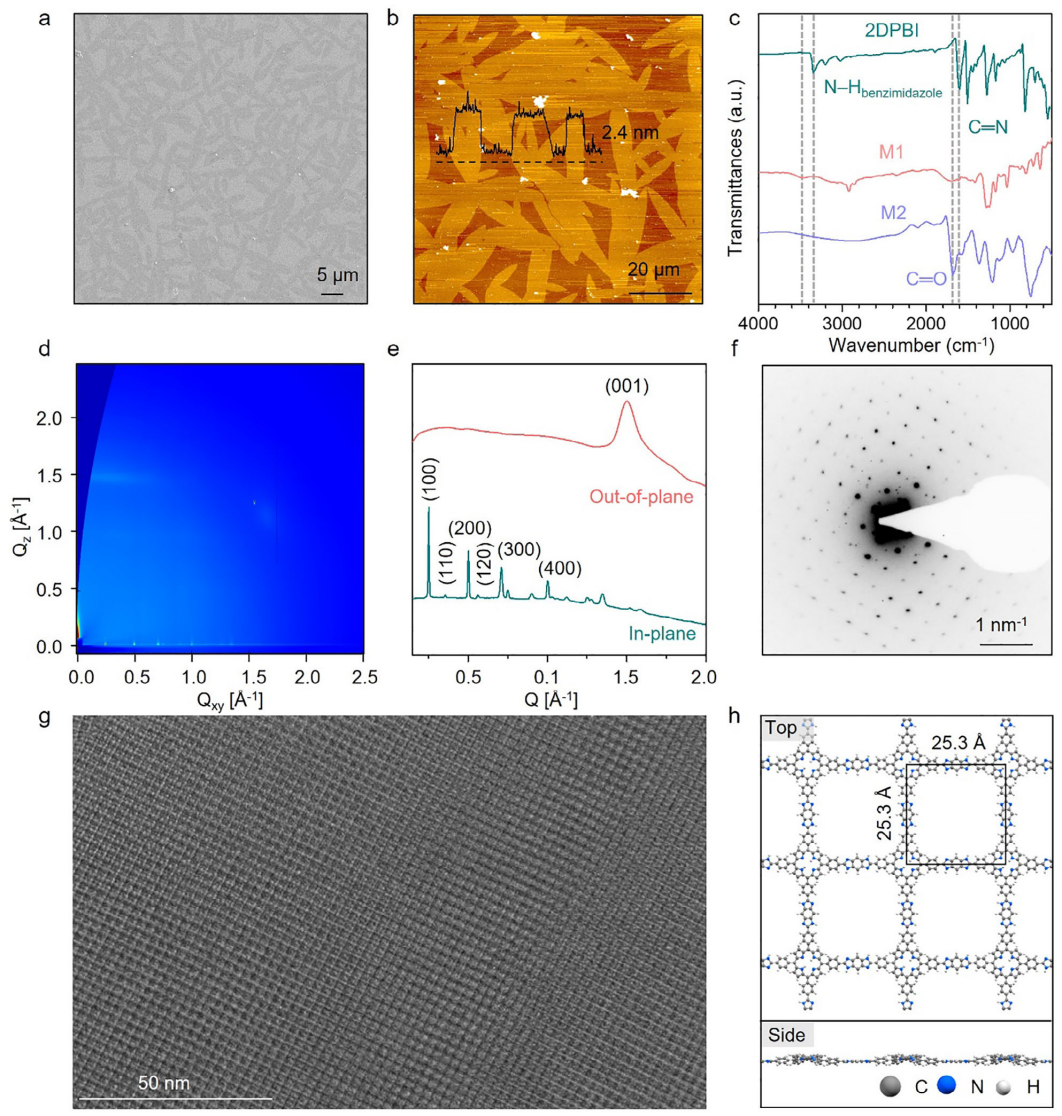
Morphological and structural characterizations of the 2DPBI film. a) FE‐SEM image of the 2DPBI film showing uniform coverage. b) AFM image revealing the few‐layer structure of the 2DPBI. c) ATR‐FTIR spectroscopy of 2DPBI indicates the presence of hydrogen‐bonded N─H within the 1,3‐diazole (imidazole) ring, with a new band emerging at 1628 cm^−1^, attributed to the C═N vibrations of the 1,3‐diazole (imidazole) ring structure. d) 2D GIWAXS pattern of the 2DPBI film. e) Experimental in‐plane (near *Q_Z_* = 0) and out‐of‐plane (near *Q_xy_* = 0) projections. f) SAED pattern of the transferred 2DPBI film demonstrating a very high degree of crystallinity. g) HR‐TEM image reveals a highly ordered square lattice with a lattice parameter of 25.1 Å. h) Theoretically calculated structures of 2DPBI along the in‐plane and out‐of‐plane directions.

The synthesized 2DPBI film, covering an area of ≈28 cm^2^, was meticulously transferred from the water surface to various substrates for detailed morphological and structural analyses. As shown in **Figure** **3**a, plentiful large 2DPBI crystals were observed, characterized by a uniform size distribution with domain sizes ranging from 110 to 140 µm2 (Figure S6, Supporting Information). Significantly, the size of these crystal domains exceeds those of previously reported 2DPs and layer‐stacked 2D COFs, synthesized through irreversible reactions.^[^ ^25^ ^]^ Atomic force microscopy (AFM) measurements indicated that these 2DPBI single crystals had a thickness of ≈2.4 nm with a surface roughness (Rq) of 0.43 nm, confirming their few‐layer nature (Figure 3b; Figure S6, Supporting Information). Further comparative analysis using Fourier‐transform infrared (FT‐IR) spectroscopy on the 2DPBI and its precursor monomers M1 and M2 revealed a notable shift in the N–H stretching vibration, moving from a broad peak at 3465 cm^−1^, which is indicative of free N–H groups, to 3325 cm^−1^, characteristic of hydrogen‐bonded N–H within the 1,3‐diazole (imidazole) ring.^[^ ^26^ ^]^ Simultaneously, pronounced new bands emerged at 1628 cm^−1^, attributable to the C═N vibrations in the 1,3‐diazole (imidazole) ring structure.^[^ ^26^ ^]^ The diminished intensity of the C═O band at 1697 cm^−1^ suggests the complete conversion to 1,3‐diazole (imidazole) linkages (Figure 3c). The N1s spectrum obtained from X‐ray photoelectron spectroscopy (XPS) showed peaks at binding energies of 400.2 and 398.1 eV, which were attributed to the amine and imine nitrogen atoms of the 1,3‐diazole linkage, respectively. Raman spectroscopy further identified prominent vibrational bands in the 1400–1650 cm^−1^ range (γ region), with the most intense peaks observed at 1480 cm^−1^ and 1617 cm^−1^, attributed to benzimidazole ring stretching vibrations, along with C═C and C═N stretching modes (Figure S7, Supporting Information).

The crystallinity and lattice structure of 2DPBI were investigated through grazing‐incidence wide‐angle X‐ray scattering (GIWAXS) and high‐resolution transmission electron microscopy (HR‐TEM) analyses. The GIWAXS patterns displayed a series of distinct Bragg spots near *Q_Z_* = 0, signifying superior crystallinity on a macroscopic scale (Figure S8, Supporting Information). In‐plane peaks at *Q_xy_* = 0.25 and 0.50 Å^−1^ correspond to the (100) and (200) Bragg reflections from a square lattice, where the lattice constants *a* and *b* are both 25.1 Å (Figure 3d,e). These findings closely align with the lattice structure of 2DPBI predicted by density functional theory (DFT) calculations (Figure S9, Supporting Information). Additionally, the detection of an intense arc at 1.51 Å^−1^ suggests π–π stacking along the c‐axis, with an interlayer spacing of ≈4.15 Å (Figure 3e). At the microscopic level, selected‐area electron diffraction (SAED) and HR‐TEM images demonstrate that each 2DPBI flake constitutes a single crystal, devoid of any detectable amorphous regions. However, lattice defects and grain boundaries were observed in overlapping regions of the crystals (Figure S10, Supporting Information). As illustrated in Figure 3f, the presence of a square unit cell with nearest reflections at 0.40 nm^−1^ further corroborates an AA‐inclined stacking model for 2DPBI (Figure S9, Supporting Information). HR‐TEM images reveal a highly ordered square lattice with a lattice parameter of 25.1 Å, showing an excellent agreement between the experimental and simulated structures (Figure 3g,h). As shown in optical microscopy images (Figure S11, Supporting Information), the morphology of 2DPBI thin films was retained after immersion in strong acidic and basic solutions, demonstrating high chemical stability attributed to the irreversible imidazole linkages.

### 2.4 Charge Transport and Photodetection in 2DPBI

**Figure 4:**
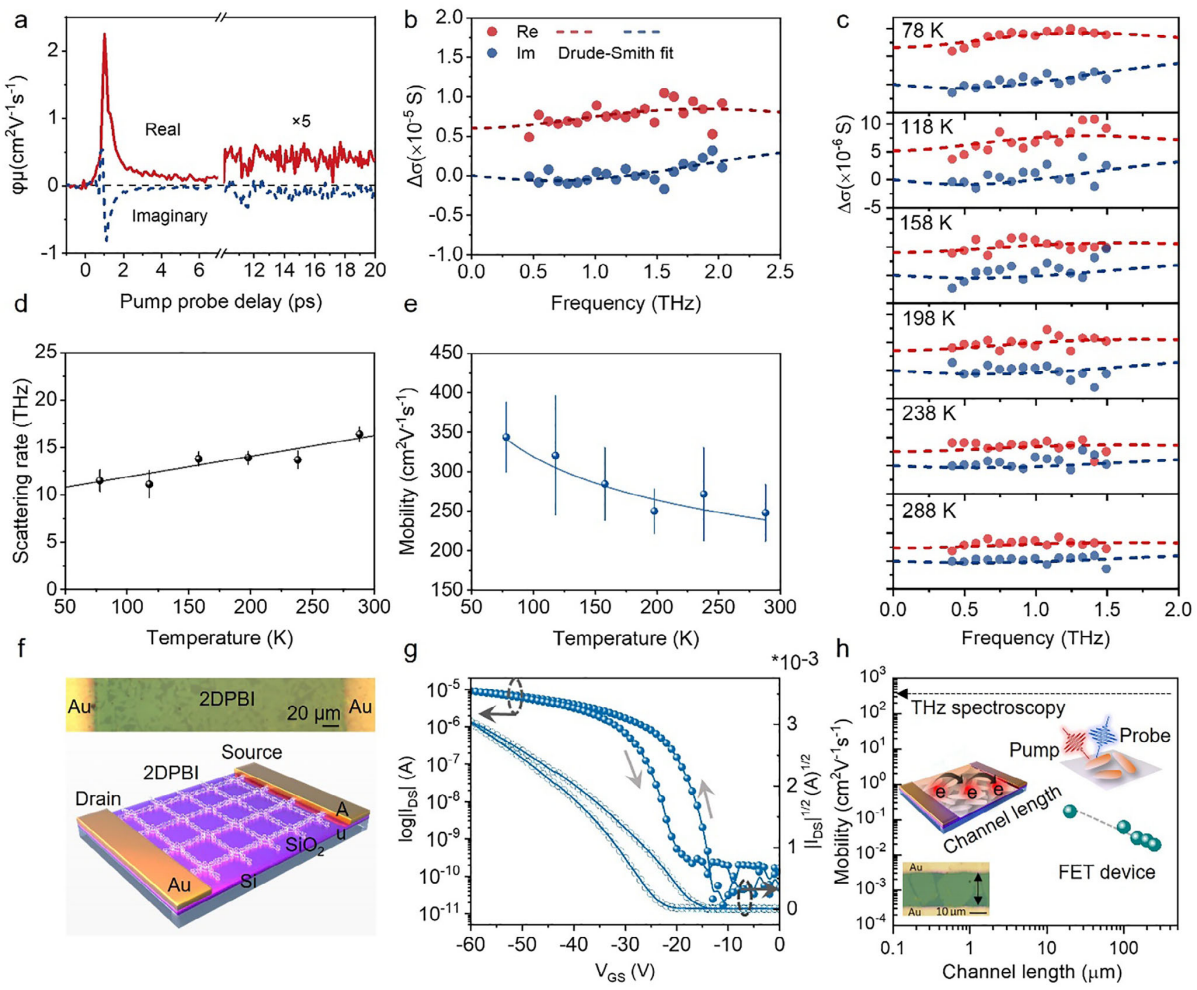
Time‐ and frequency‐resolved complex photoconductivity by optical pump‐THz probe spectroscopy. a) Real and imaginary parts of the photoconductivity dynamics of 2DPBI thin film, excited by a 400 nm femtosecond laser pulse. b) Room‐temperature complex photoconductivity spectra of 2DPBI thin film recorded 10 ps after excitation. The red and blue dots represent the experimental data, and the dashed lines are the fits by the Drude‐Smith model. c) Complex photoconductivity spectra and Drude‐Smith fits at different temperatures from 78 to 288 K. d) The inferred scattering rate as a function of temperature. The black dots are the experimental data, while the solid line is a guide to the eye. e) The charge mobility as a function of temperature. The blue dots represent the experimental data, and the solid line is the fit of a power law *T^β^* with *β* of −0.27. f) The schematic device structure of the bottom‐gate, top‐contact (BGTC) transistor configuration employed for the measurements. g) Current versus gate voltage sweep curves at an applied drain voltage of −60 V for the long channel (300 µm) 2DPBI transistors. The measurements were performed in the dark. h) FET devices with different channel lengths have revealed that mobility increases as the channel length decreases.

Next, we use optical pump‐THz probe (OPTP) spectroscopy as a contact‐free all‐optical approach to investigate the charge transport properties of 2DPBI films. We photo‐inject charge carriers into the sample by above‐bandgap excitation with 400 nm light pulses (at an excitation fluence of 91 µJ cm^−2^). A single‐cycle THz pulse with ≈1 ps duration interacts with the photoinjected carriers, giving rise to THz attenuation and phase shift. By recording the transmitted THz electric field, we access short‐range charge transport properties for the sample of interest^[^ ^27^ ^]^ thanks to the transient nature of the THz electric field. The relative attenuation of the THz electric field *E*(*t*) is proportional to the real part (*Re*) of the complex photoconductivity *Δσ*, *Δσ_Re_*, while the phase‐shift is related to the imaginary part (*Im*) of the photoconductivity *Δσ_Im_* (typically observed for excitons).^[^ ^27^ ^]^ **Figure** **4**a shows the dynamics of *Δσ_Re_* and *Δσ_Im_* within 20 ps. The relatively large value for *Δσ_Re_* compared to *Δσ_Im_* illustrates that the response is dominated by free charge carriers.

We note that, in contrast to most reported 2DPs and COFs, where *Δσ_Re_* decays to ≈zero within a few ps due to rapid charge localization (e.g., by charge trapping or exciton formation),^[^ ^28^ ^]^ 2DPBI exhibits a relatively long‐lived, positive *Δσ_Re_* for well over 20 ps. This suggests that the remaining charge carriers following the initial decay survive as free carriers for a long time. Furthermore, the frequency‐resolved photoconductivity spectrum measured 10 ps after photoexcitation (Figure 4b) is characterized by a finite, positive *Δσ_Re_* ‐dominated signal and a near‐zero *Δσ_Im_*, consistent with free carrier behavior. The dispersion can be well fitted by the Drude‐Smith (DS) model, which describes spatially confined free carrier transport following:^[^ ^27^, ^29^ ^]^

(1) $$\sigma_{DS}=\frac{\varepsilon_{0}\omega_{p}^{2}\tau }{1-i\omega \tau }\;\left(1+\frac{c}{1-i\omega \tau }\right)$$

Here, ε_0_ is the vacuum permittivity, ω_*p*_ is the plasma frequency, and τ is the momentum scattering time. The extent of the confinement effect limiting the long‐range *dc* transport is evaluated by the parameter *c* (−1  ≤ *c* ≤  0). For *c*  =  0, the model reverts to the classical Drude model, where scattering events completely randomize the momentum, while for *c* approaching −1, the carriers undergo preferential backscattering. Combined with the charge scattering time (τ  = 62 ± 8 fs), the reduced electron‐hole effective mass (m*) of 0.171*m*_0_, and the inferred *c*  = −0.62 ± 0.04, 2DPBI exhibits superior charge transport at room temperature with an outstanding charge mobility in the *dc* limit: µ_*dc*_ = *e*τ/m* (1 + *c_p_*)= 240 ± 38 cm^2^ V^−1^ s^−1^. The estimated mobility exceeds previously reported values for metal‐free 2DPs and COFs (e.g., 165 cm^2^ V^−1^ s^−1^ for imine‐linked TPB‐TFB COF).^[^ ^30^ ^]^

To further explore the charge transport mechanism, we measured temperature‐dependent frequency‐resolved photoconductivity spectra (Figure 4c). By fitting the data to the DS model, we extracted the scattering rate (the inverse of the scattering time) and parameter *c* at different temperatures, thereby obtaining the *dc* mobility (µ  = *e* · τ · (1 + *c*)/m* ) as a function of temperature. With decreasing temperature, the reduced phonon population results in a clear reduction in the scattering rate (Figure 4d), leading to a pronounced increase in charge mobility from ≈240 cm^2^ V^−1^ s^−1^ at room temperature to ≈350 cm^2^ V^−1^ s^−1^ at 77K, as shown in Figure 4e. These observations are in line with the band‐like transport mechanism where the charge carriers are strongly delocalized and limited by phonon scattering at finite temperatures. By phenomenologically fitting the *T*‐dependent charge mobility using a power law µ∝*T* ^β^, we infer a decay constant *β* of ‐0.27. Calculations using Boltzmann transport theory under the constant relaxation time approximation (CRTA) are consistent with experimental observations (for a detailed discussion, see Figures S12–S21, Supporting Information).

Field effect transistor (FET) devices were fabricated by transferring the 2DPBI thin films onto a highly n‐doped silicon (Si) substrate with a 300 nm SiO_2_ dielectric layer (for detailed device fabrication procedures, see Figure S22, Supporting Information). A bottom‐gate top‐contact (BGTC) architecture was employed, wherein Si was used as the gate contact and evaporated gold (Au) was used as the top source‐drain contact (schematically shown in Figure 4f). To evaluate charge carrier mobility, the devices were swept across the gate voltage (V_GS_) with an applied drain‐source voltage (V_DS_) of −60 V, and the transfer characteristics thus obtained are shown in Figure 4g,h. We measured FET devices with varying channel lengths, from 300 to 20 µm, and observed that the mobility increased as the channel length decreased. Specifically, the mobility reached 0.17 ± 0.008 cm^2^ V^−1^ s^−1^ for devices with a channel length of 20 µm (Figure S23, Supporting Information). In THz photoconductivity measurements, the transient terahertz field, with a ps duration, drives charge carriers over distances of approximately tens of nanometers.^[^ ^31^ ^]^ Thus, terahertz spectroscopy provides information on local, intra‐flake charge transport in the 2DPBI film. FET electrical transport studies measure long‐range charge carrier conductance over macroscopic distances between electrodes under a DC bias. The 2DPBI film exhibits a relatively large domain size, as shown in Figure 3b, which promotes charge transport (Figure 4h) even in devices with large channels (300 µm), an achievement that has not been reported to date for 2DP and COF‐based materials.

**Figure 5:**
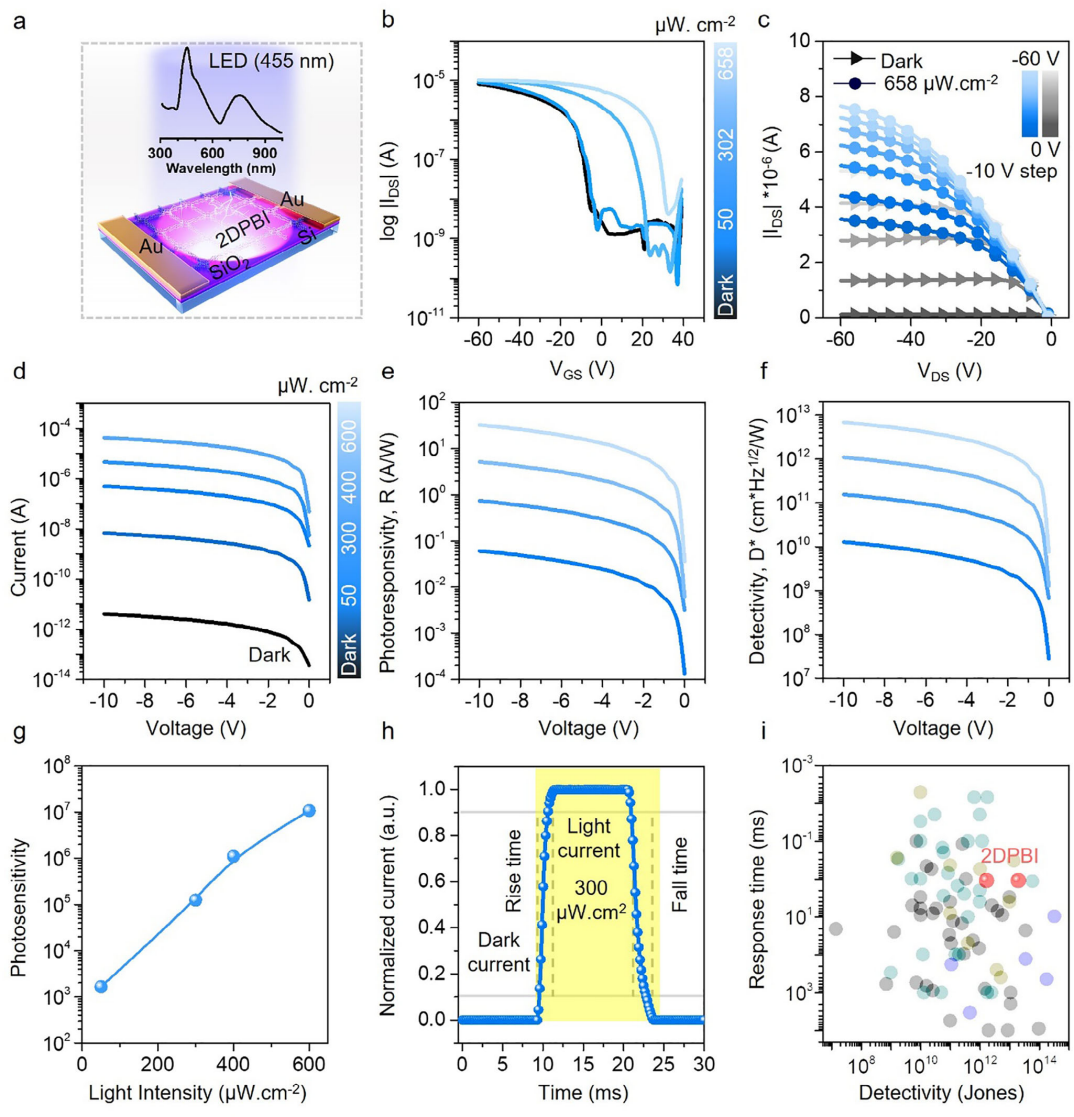
Photodetection with 2DPBI phototransistors (a) shows the schematic device structure of the bottom‐gate, top‐contact (BGTC) transistor configuration employed for the measurements along with the absorption spectrum of 2DPBI. b) Electrical transfer characteristics for different illumination intensities of a long channel 2DPBI transistor. The channel length and width were 300 µm and 11000 µm, respectively. c) Phototransistor output characteristics observed for these long channel devices in the dark and under the influence of 455 nm blue light. d) Current‐voltage characteristics of the 2DPBI film with a 20 µm channel length at V_GS_ = 0 V, indicating photodetection using a 455 nm light source, followed by the calculated (e) photoresponsivity and (f) detectivity under varying illumination intensities of the light source. g) Measured photosensitivity at V_GS_ = 0 V and V_DS_ = ‐10 V. h) Response time of the photodetector under a 455 nm light source with an illumination intensity of 300 µW cm^−^^2^. i) Comparison plot of detectivity and response time for different materials: gray represents standalone 2D materials, cyan represents graphene‐based heterostructure materials (including COF and MOF), green represents MoS_2_‐based heterostructure materials, blue represents other 2D materials combined with small organic molecule heterostructures, and red represents standalone 2DPBI with detectivity measurements based on both noise current and dark current.

Benefitting from strong optical absorption, efficient electron‐hole charge separation, and excellent charge transport properties, including high mobility, we further studied the optoelectronic performance of the phototransistor devices. With the absorption maxima of 2DPBI ≈450 nm (as shown in **Figure** **5**a), the thin film responds to illumination from a blue‐light source, as demonstrated in the previously described transistors with their transfer and output characteristics shown in Figure 5b,c. As the intensity of blue light increases, we observe that the turn‐on voltage shifts toward more positive values. Additionally, at V_GS_ = 0 V, from the transfer characteristics shown in Figure 5c, a photo‐current gain of four orders of magnitude was obtained at an illumination intensity of 658 µWcm^−2^.

For photodetection‐based devices, it is crucial to evaluate the optical characteristics alongside the electrical characteristics. While transfer curves of transistors are often used to access optical figures of merit, it is necessary to study the photodetection with minimum influence of an external electric field (from the gate contact), which can modulate the actual effect of electrons/holes. Therefore, the optical figures of merit, photosensitivity (P), photoresponsivity (R) and detectivity (D*) of the devices, were investigated using the *I*–*V* curves obtained with 0 V applied at the gate contact. The *I–V* graph was shown in Figure 5d. In the dark, the measured current values were in the range of 10^−12^A, indicating that the device is in the off‐state. When an illumination intensity of 50 µWcm^−2^ was applied, the current increased by three orders of magnitude. This confirmed that the 2DPBI film is sensitive to blue light illumination as low as 50 µWcm^−2^. At a maximum illumination intensity of 600 µWcm^−2^, a substantial seven‐order‐of‐magnitude improvement in the current measured was observed. We attribute this to the increased charge generation upon increased illumination intensity (Figures S24–S28, Supporting Information). The calculated P, R and D* values for these measurements are presented in Figure 5e‐g. As P is linked to the current gain, Figure 5g reveals a similar behavior observed in Figure 5d. The response time of the photodetector under a 455 nm light source with an illumination intensity of 300 µWcm^−2^ is measured with a rise time of 1.1 ms and a fall time of 1.8 ms (Figure 5h; Figure S29, Supporting Information). The P and R stood at 1.08 × 10^7^ and 32 AW^−1^, respectively, at an illumination intensity of 600 µWcm^−2,^ which are the best‐reported values for standalone few‐layer 2D material‐based devices to date,^[^ ^2^, ^10^ ^]^ as shown in the comparison plot in Figure 5i. In addition, the figures of merit are plotted against the gate voltage in Figures S24–S28 (Supporting Information) for reference. Furthermore, the calculated specific detectivity values of 7.1 × 10^12^ Jones and 2.0 × 10^13^ Jones, based on noise current and dark current measurements respectively, are higher than previously reported values in the literature, including those of conventional Si‐based optoelectronic devices (for detailed comparison, see Tables S1–S3, Supporting Information).^[^ ^8^, ^10^ ^]^

## 3 Conclusion

In summary, our study presents the successful synthesis of 2DPBI, demonstrating high charge carrier mobility and excellent photoresponse performance. The unique π‐conjugated porphyrin motif and 1,3‐diazole linkage in 2DPBI result in notable optoelectronic properties, including a narrow band gap of 1.18 eV, a high resonant absorption coefficient, and an impressive THz charge carrier mobility of ≈240 cm^2^ V^−1^ s^−1^. Our findings, supported by terahertz spectroscopy, reveal a band‐like transport mechanism. Furthermore, the long‐channel transistors based on 2DPBI exhibit outstanding performance metrics with an on/off ratio exceeding 10^8^, a photosensitivity of 1.08 × 10^7^, photoresponsivity of 32 AW^−1^, and detectivity values of 7.1 × 10^12^ Jones and 2.0 × 10^13^ Jones (based on noise current and dark current measurements, respectively). These results establish 2DPBI as a promising candidate for next‐generation multifunctional optoelectronic devices, offering a unique combination of high charge carrier mobility and robust photoresponsive characteristics. To advance toward practical applications, future efforts will focus on enhancing large‐area uniformity and long‐range order through tailored surfactant design and precise interfacial control to enable continuous single‐crystalline thin films.

## 4 Experimental Section

### Synthesis of 2DPBI Through the Surfactant Monolayer Assisted Interfacial Synthesis (SMAIS) Method

Milli‐Q water (40 mL) was injected into a beaker (80 mL, diameter = 6 cm) to form a static air‐water interface. Then, 15 µL of CTAB (1 mg mL^−1^ in chloroform) was spread onto the surface. The solvent was allowed to evaporate for 10 min, and then M1 (0.6 mL, 1 mg mL^−1^ in 0.05 M LiOH aqueous solution) was gently added to the water subphase using a syringe. After 1 h, M2 (0.8 mL, 1 mg mL^−1^ in 0.30 M HCl aqueous solution) was gradually added to the water subphase with a syringe. The reaction was then kept undisturbed at room temperature for three days. Upon visual inspection, the reaction results in the formation of a film on the surface of the water. The resulting film was collected from the water surface onto the substrate by the horizontal dipping method.

### General Characterization

Optical microscopy (Zeiss), AFM (Bruker Multimode 8 HR), and TEM (Zeiss, Libra 200 KV) were used to investigate the morphology and structure of the samples. Thin films were deposited on a Si substrate for SEM measurements and on copper grids for TEM measurements. All the optical microscopy and AFM images were recorded on a 300 nm SiO_2_/Si substrate. UV–vis absorption spectra were recorded on a UV–vis–NIR spectrophotometer Cary 5000 device at room temperature on a quartz glass substrate. Photoluminescence spectra were measured on the PerkinElmer fluorescence spectrometer LS 55. ATR‐FTIR was performed on a Tensor II system (Bruker) with an attenuated total reflection unit, and the samples were prepared by depositing the thin films onto a copper foil. Time‐dependent surface‐pressure measurements were carried out by the Langmuir‐Blodgett trough (KSV NIMA, Finland). The trough was equipped with a platinum Wilhelmy plate, a Teflon dipper, and a pair of Delrin barriers. ^1^H NMR spectra were recorded at room temperature with a Bruker Avance III HD 300 spectrometer operating at 300 MHz. DMSO‐d_6_ (DMSO, dimethylsulfoxide) was used as the solvent. The high‐resolution matrix‐assisted laser desorption/ionization time‐of‐flight (MALDI‐TOF) mass spectrometry was performed on a Bruker Autoflex Speed MALDI‐TOF MS using trans‐2‐[3‐(4‐tert‐butylphenyl)‐2‐methyl‐2‐propenylidene] malononitrile (DCTB) or dithranol as matrix.

### Grazing‐Incidence Wide‐Angle X‐Ray Scattering

GIWAXS measurements were performed at beamline P08 at the German Electron Synchrotron (Deutsches Elektronen‐Synchrotron) DESY, Hamburg, Germany. The energy of the beam was 18 keV, and the dimensions of the beam were 100 µm (vertically) and 400 µm (horizontally). The images were recorded using a Perkin Elmer 1620 area detector which was placed 707 mm behind the sample. The sample‐to‐detector distance and the position of the beam on the detector were verified using a lanthanum hexaboride calibration standard. The incidence angle of the beam was 0.1° and the samples were exposed for 30 s. Data correction and analysis were performed using WxDiff.

### Optical Pump‐THz Probe (OPTP) Spectroscopy

The time‐ and frequency‐resolved photoconductivity of 2D‐PBI was performed by the OPTP spectroscopy. A commercial regeneratively amplified and mode‐locked Ti:sapphire femtosecond laser system was employed to deliver ultrashort laser pulses with a duration of ≈ 50 fs, a central wavelength of 800 nm, and a repetition rate of 1 kHz. A pair of (110)‐oriented zinc telluride (ZnTe) crystals were used to generate and detect THz radiation covering the frequency range of ≈0.4–2.0 THz via optical rectification and electro‐optical sampling methods, respectively. In the terahertz (THz) spectroscopy measurements, the optical pumping pulse duration was 100 femtoseconds. The 2DPBI film supported on the fused silica substrate was photoexcited using 400 nm laser pulses obtained by frequency‐doubling the fundamental 800 nm laser pulses in a *β*‐barium borate crystal. The real and imaginary parts of the time‐resolved photoconductivity were measured by recording the pump‐induced changes at the peak of the THz waveform and at the zero crossing after the peak, respectively, while adjusting the time delay between the pump and the THz probe. The frequency‐resolved photoconductivity at a certain pump‐probe delay was obtained by recording entire THz waveforms with and without photoexcitation and applying fast Fourier Transform and the thin‐film approximation. The room‐temperature measurements were performed in a dry N_2_‐purged environment and the low‐temperature measurements were conducted in a cryostat under vacuum conditions.

### Phototransistor Device Measurements

Measurements were done utilizing a Keithley 4200A‐SCS Parameter Analyzer controlled by the SweepMe! software in a dark room at ambient conditions. Prior to the measurements, thermally evaporated, 50 nm thick Au (source‐drain) contacts were deposited on the 2DPBI films transferred onto a Si/SiO_2_ (300 nm) substrate. The contact deposition was performed under high vacuum conditions of 10^−7^ mbar with a deposition velocity of 1.5 Å s^−1^ using a shadow mask. The effective field‐effect mobility, µ of devices in saturation was calculated using the equation:

(2) $$\mu =\frac{2L}{WC_{i}}\;{\left(\frac{\partial \sqrt{I_{DS}}}{\partial V_{GS}}\right)}^{2}$$

from the fitted linear slopes between the threshold voltage, V_TH_, and maximum gate voltage, V_GS_. Here, C_i_ is the capacitance per unit area of the dielectric, W is the channel width, and L is the channel length. The V_TH_ was obtained from the intercept of the linear fit of the $\sqrt{I_{DS}}$ versus V_GS_ curve and the x‐axis. Blue (455 nm) light exposure was achieved using LED M455L4 from Thorlabs. The light intensity was measured using a reference Si photodiode (S120VC) and a power meter (PM100D) from Thorlabs. During the phototransistor characterization experiments, the illumination intensity was controlled by varying the source power.

### Theoretical Calculations

For DFTB calculations, DFTB+ code was used.^[^ ^32^ ^]^ Calculations were performed with 3ob parametrization set^[^ ^33^ ^]^ without using self‐consistent charges. In geometry optimizations, a 5 × 5 × 1 k‐mesh was used for Brillouin zone integration and maximum force tolerance was set to 10^−8^ a.u. Charge density calculations were performed by using the Vienna ab‐initio Simulation Package (VASP).^[^ ^34^ ^]^ The Perdew–Burke–Ernzerhof (PBE)^[^ ^35^ ^]^ functional was used to describe electron exchange and correlation. The DFT‐D2 method of Grimme^[^ ^36^ ^]^ was used to describe van der Waals forces in layered structures. The kinetic energy cut‐off for plane‐wave expansion was set to 500 eV and the energy was minimized until its variation in the following steps became 10^−5^ eV. The Gaussian smearing method was employed for the total energy calculations and the width of the smearing was chosen as 0.05 eV. Total Hellmann‐Feynman forces in the until were reduced to 10^−3^ eV/Å. A 3 × 3 × 1 Γ‐centered mesh was used for the Brillouin zone integration. For the monolayer, a vacuum space of 15 Å was incorporated for the confined *z‐*direction. 2000 grid points were used for *lm*‐decomposed density of states evaluation. To investigate the non‐parabolicity in electronic bands, effmass Python package^[^ ^37^ ^]^ and in‐house python scripts were used. Phonopy code^[^ ^38^ ^]^ was used to calculate the phonon band structure. A 3 × 3 × 1 supercell was used. The chosen displacement amplitude was 0.005 Å. The geometric symmetry tolerance was set to 10^−5^. The transport properties of monolayer 2DPBI were calculated by solving the Boltzmann equation within the constant relaxation time approximation (CRTA) as implemented in DFTBephy package.^[^ ^39^ ^]^ A 3 × 3 × 1 supercell and a 50 × 50 × 1 k‐mesh were used for conductivity calculations. The transport properties were also evaluated within the rigid‐band model as implemented in the BoltzTraP2 code.^[^ ^40^ ^]^ The ground state Kohn–Sham eigenvalues, obtained from VASP calculations, were interpolated onto a 250 × 250 × 1‐grid in the irreducible Brillouin zone. The experimental relaxation time values were used.

## Conflict of Interest

The authors declare no conflict of interest.

## Supporting information



Supporting Information

## Data Availability

The data that support the findings of this study are available from the corresponding author upon reasonable request.

## References

[1] a) Y. Chen , M. Nazhamaiti , H. Xu , Y. Meng , T. Zhou , G. Li , J. Fan , Q. Wei , J. Wu , F. Qiao , Nature 2023, 623, 48; 37880362 10.1038/s41586-023-06558-8 PMC10620079

[2] a) S. Zhang , R. Chen , D. Kong , Y. Chen , W. Liu , D. Jiang , W. Zhao , C. Chang , Y. Yang , Y. Liu , Nat. Nanotechnol. 2024, 19, 1323; 38965348 10.1038/s41565-024-01707-0

[3] a) G. Zhao , J. Liu , Q. Meng , D. Ji , X. Zhang , Y. Zou , Y. Zhen , H. Dong , W. Hu , Adv. Electron. Mater. 2015, 1, 1500071;

[4] a) A. Dodda , D. Jayachandran , A. Pannone , N. Trainor , S. P. Stepanoff , M. A. Steves , S. S. Radhakrishnan , S. Bachu , C. W. Ordonez , J. R. Shallenberger , Nat. Mater. 2022, 21, 1379; 36396961 10.1038/s41563-022-01398-9

[5] a) S. V. Morozov , K. S. Novoselov , M. I. Katsnelson , F. Schedin , D. C. Elias , J. A. Jaszczak , A. K. Geim , Phys. Rev. Lett. 2008, 100, 016602; 18232798 10.1103/PhysRevLett.100.016602

[6] a) S. Kim , A. Konar , W.‐S. Hwang , J. H. Lee , J. Lee , J. Yang , C. Jung , H. Kim , J.‐B. Yoo , J.‐Y. Choi , Nat. Commu. 2012, 3, 1011; 10.1038/ncomms2018 22910357

[7] a) J. Han , F. Wang , S. Han , W. Deng , X. Du , H. Yu , J. Gou , Q. J. Wang , J. Wang , Adv. Funct. Mater. 2022, 32, 2205150;

[8] a) F. P. García de Arquer , A. Armin , P. Meredith , E. H. Sargent , Nat. Rev. Mater. 2017, 2, 16100;

[9] a) J. Han , J. Wang , M. Yang , X. Kong , X. Chen , Z. Huang , H. Guo , J. Gou , S. Tao , Z. Liu , Adv. Mater. 2018, 30, 1804020; 10.1002/adma.201804020 30276886

[10] a) A. Chetia , J. Bera , A. Betal , S. Sahu , Mater. Today Commun. 2022, 30, 103224;

[11] a) K. Sotthewes , R. Van Bremen , E. Dollekamp , T. Boulogne , K. Nowakowski , D. Kas , H. J. W. Zandvliet , P. Bampoulis , J. Phys. Chem. C. 2019, 123, 5411; 10.1021/acs.jpcc.8b10971 PMC6410613 30873255

[12] a) T. He , H. Ma , Z. Wang , Q. Li , S. Liu , S. Duan , T. Xu , J. Wang , H. Wu , F. Zhong , Nat. Photon. 2024, 18, 60;

[13] a) G. Bian , J. Yin , J. Zhu , Small 2021, 17, 2006043; 10.1002/smll.202006043 33624949

[14] a) X. Feng , A. D. Schlüter , Angew. Chem., Int. Ed. 2018, 57, 13748; 10.1002/anie.201803456 29845730

[15] C. Wang , Z. Zhang , Y. Zhu , C. Yang , J. Wu , W. Hu , Adv. Mater. 2022, 34, 2102290. 10.1002/adma.202102290 35052010

[16] X. Li , P. Yadav , K. P. Loh , Chem. Soc. Rev. 2020, 49, 4835. 32490450 10.1039/d0cs00236d

[17] a) Y. Xiong , Q. Liao , Z. Huang , X. Huang , C. Ke , H. Zhu , C. Dong , H. Wang , K. Xi , P. Zhan , Adv. Mater. 2020, 32, 1907242; 10.1002/adma.201907242 31990415

[18] A. M. Evans , M. J. Strauss , A. R. Corcos , Z. Hirani , W. Ji , L. S. Hamachi , X. Aguilar‐Enriquez , A. D. Chavez , B. J. Smith , W. R. Dichtel , Chem. Rev. 2021, 122, 442. 34852192 10.1021/acs.chemrev.0c01184

[19] L. Cusin , H. Peng , A. Ciesielski , P. Samorì , Angew. Chem., Int. Ed. 2021, 133, 14356. 10.1002/anie.202016667 33491860

[20] a) O. I. Dosunmu , D. D. Cannon , M. K. Emsley , B. Ghyselen , J. Liu , L. C. Kimerling , M. S. Unlu , IEEE J. Sel. Top. Quantum Electron. 2004, 10, 694;

[21] N. Li , P. Mahalingavelar , J. H. Vella , D.‐S. Leem , J. D. Azoulay , T. N. Ng , Mater. Sci. Eng. R Rep. 2021, 146, 100643.

[22] X. Gong , M. Tong , Y. Xia , W. Cai , J. S. Moon , Y. Cao , G. Yu , C.‐L. Shieh , B. Nilsson , A. J. Heeger , Science 2009, 325, 1665. 19679770 10.1126/science.1176706

[23] a) Q. Wang , W. M. Campbell , E. E. Bonfantani , K. W. Jolley , D. L. Officer , P. J. Walsh , K. Gordon , R. Humphry‐Baker , M. K. Nazeeruddin , M. Grätzel , J. Phys. Chem. B. 2005, 109, 15397; 16852953 10.1021/jp052877w

[24] a) A. Prasoon , X. Yu , M. Hambsch , D. Bodesheim , K. Liu , A. Zacarias , N. N. Nguyen , T. Seki , A. Dianat , A. Croy , Nat. Commu. 2023, 14, 8313; 10.1038/s41467-023-44129-7 PMC10721922 38097633

[25] a) E. Jin , M. Asada , Q. Xu , S. Dalapati , M. A. Addicoat , M. A. Brady , H. Xu , T. Nakamura , T. Heine , Q. Chen , Science 2017, 357, 673; 28818940 10.1126/science.aan0202

[26] K. C. Ranjeesh , R. Illathvalappil , S. D. Veer , J. Peter , V. C. Wakchaure , K. V. R. Goudappagouda , S. Kurungot , S. S. Babu , J. Am. Chem. Soc. 2019, 141, 14950. 31510740 10.1021/jacs.9b06080

[27] R. Ulbricht , E. Hendry , J. Shan , T. F. Heinz , M. Bonn , Rev. Mod. Phys. 2011, 83, 543.

[28] A. Tries , S. Osella , P. Zhang , F. Xu , C. Ramanan , M. Kläui , Y. Mai , D. Beljonne , H. I. Wang , Nano Lett. 2020, 20, 2993. 32207957 10.1021/acs.nanolett.9b04816 PMC7311082

[29] N. V. Smith , Phys. Lett. A. 1968, 26, 126.

[30] S. Fu , E. Jin , H. Hanayama , W. Zheng , H. Zhang , L. Di Virgilio , M. A. Addicoat , M. Mezger , A. Narita , M. Bonn , J. Am. Chem. Soc. 2022, 144, 7489. 35420808 10.1021/jacs.2c02408 PMC9052747

[31] W. Zheng , B. Sun , D. Li , S. M. Gali , H. Zhang , S. Fu , L. Di Virgilio , Z. Li , S. Yang , S. Zhou , Nat. Phys. 2022, 18, 544.

[32] B. Hourahine , B. Aradi , V. Blum , F. Bonafe , A. Buccheri , C. Camacho , C. Cevallos , M. Y. Deshaye , T. Dumitrică , A. Dominguez , J. Chem. Phys. 2020, 152, 124101. 32241125 10.1063/1.5143190

[33] M. Gaus , A. Goez , M. Elstner , J. Chem. Theory Comput. 2013, 9, 338. 26589037 10.1021/ct300849w

[34] a) G. Kresse , J. Hafner , Phys. Rev. B. 1993, 47, 558; 10.1103/physrevb.47.558 10004490

[35] J. P. Perdew , K. Burke , M. Ernzerhof , Phys. Rev. Lett. 1996, 77, 3865. 10062328 10.1103/PhysRevLett.77.3865

[36] S. Grimme , J. Comp. Chem. 2006, 27, 1787. 16955487 10.1002/jcc.20495

[37] L. D. Whalley , J. Open Source Softw. 2018, 3, 797.

[38] A. Togo , I. Tanaka , Scr. Mater. 2015, 108, 1.

[39] A. Croy , E. Unsal , R. Biele , A. Pecchia , J. Comput. Electron. 2023, 22, 1231.

[40] G. K. H. Madsen , J. Carrete , M. J. Verstraete , Comput. Phys. Commun. 2018, 231, 140.

