# Supplementary material for: High‐Performance Phototransistor Based on a 2D Polybenzimidazole Polymer
Source: Adv Mater. 2025 Jun 1;37(33):2505810. doi: 10.1002/adma.202505810 (PMC12369690; doi:10.1002/adma.202505810)
Supplement: Supplementary file 1 — Supporting Information [file ADMA-37-2505810-s001.pdf]

ADVANCED MATERIALS

Supporting Information

for *Adv. Mater.*, DOI 10.1002/adma.202505810

High-Performance Phototransistor Based on a 2D Polybenzimidazole Polymer

Anupam Prasoon, Preetam Dacha, Heng Zhang, Elif Unsal, Mike Hambsch, Alexander Croy, Shuai Fu, Nguyen Ngan Nguyen, Kejun Liu, Haoyuan Qi, Sein Chung, Minyoung Jeong, Lei Gao, Ute Kaiser, Kilwon Cho, Hai I. Wang, Renhao Dong, Gianaurelio Cuniberti, Mischa Bonn*, Stefan C. B. Mannsfeld* and Xinliang Feng**

Supporting Information

High-Performance Phototransistor Based on a Two-Dimensional Polybenzimidazole Polymer

Anupam Prasoon, Preetam Dacha, Heng Zhang, Elif Unsal, Mike Hambsch, Alexander Croy, Shuai Fu, Nguyen Ngan Nguyen, Kejun Liu, Haoyuan Qi, Sein Chung, Minyoung Jeong, Lei Gao, Ute Kaiser, Kilwon Cho, Hai I. Wang, Renhao Dong, Gianaurelio Cuniberti, Mischa Bonn*, Stefan C. B. Mannsfeld*, Xinliang Feng**

Materials and Methods

5-(4-carboxyphenyl)-10,15,20-(triphenyl) porphyrin (R1), 5,10,15,20-(tetra-4-aminophenyl) porphyrin (M1), and 5,10,15,20-(tetra-4-carboxyphenyl)porphyrin (M3) were obtained from PorphyChem. Other chemicals (R2, R4 - R15) and solvents, such as chloroform and THF, were obtained from PorphyChem, abcr GmbH, and Sigma-Aldrich and were used without further purification. Purified water was obtained through a Milli-Q purification system (Merck KGaA). All site-selective chemical reactions were carried out under ambient atmospheric conditions and at room temperature. The substrates used, including 300 nm SiO_2/Si wafers, quartz glass, and copper grids for TEM, were obtained from Microchemicals and Plano GmbH.

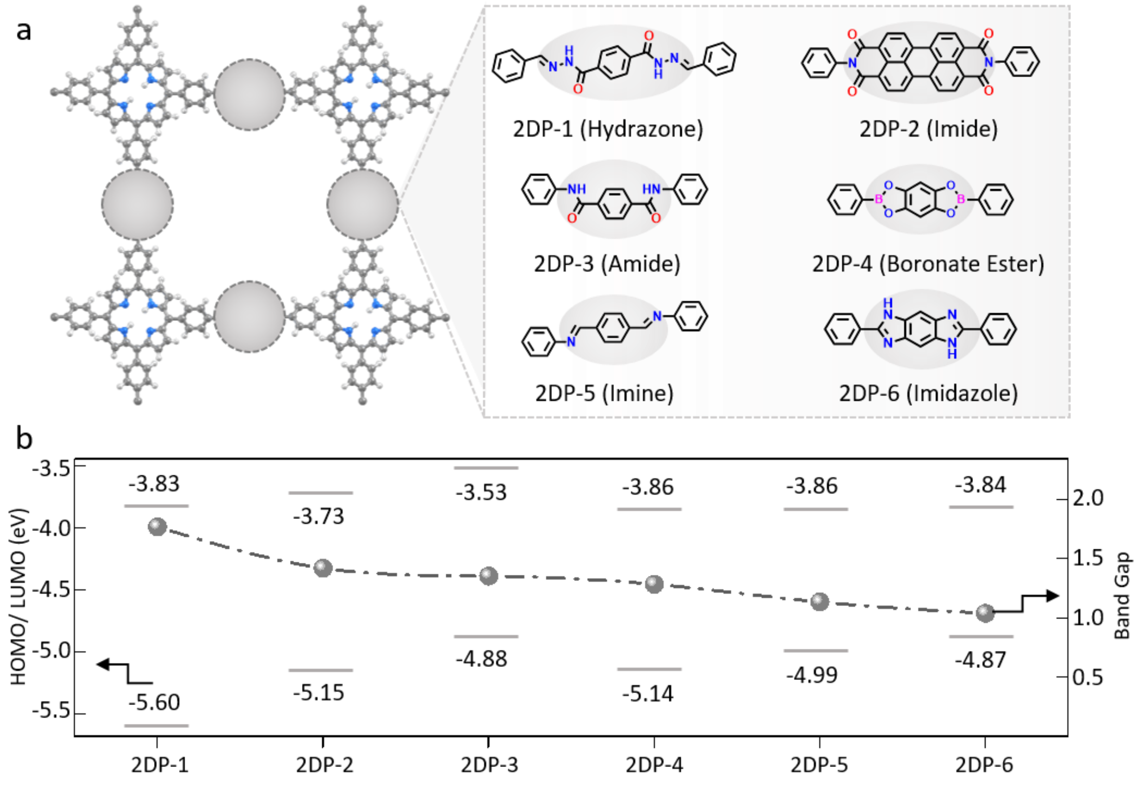

Figure S1. Design principal for Photoresponsivity in 2D Polymers **(a)** Schematic illustration of metal-free porphyrin-based 2DPs connected by various linkages, with the selection ranging from 2DP-1 to 2DP-6. **(b)** HOMO-LUMO energy levels and gaps for 2DP-1 to 2DP-6.

Porphyrin-based 2D Polymers

In addition to the monolayer 2DPBI, we investigated the electronic properties of porphyrin-based 2D polymers containing amide, boronate ester, and imine linkages. Calculations for the geometry optimization and the electronic properties were performed with DFTB+ code[1] using matsci-0-3 parametrization set[2]. Among these, 2DPBI exhibits the narrowest band gap and relatively dispersed valence bands as seen in **Supplementary Fig.2**. The valence bands of all two-dimensional polymers are characterized by the hybridization of the C-*p* and N-*p* orbitals.

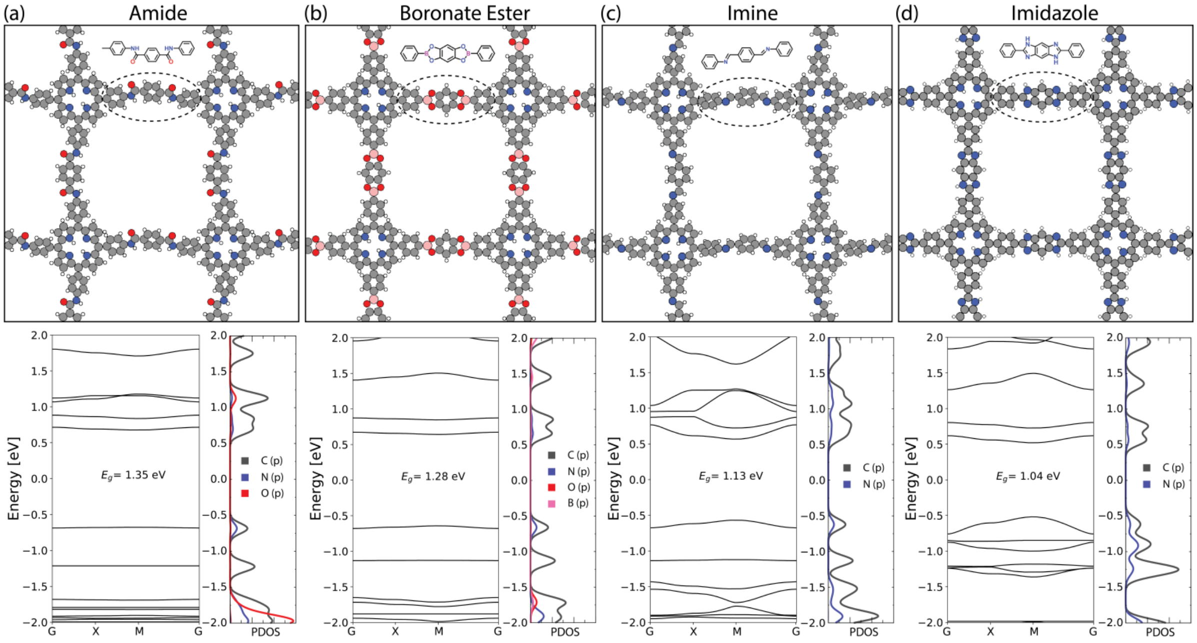

Figure S2. The calculated geometries, the electronic bands and the projected density of states of porphyrin-based 2D polymers connected by (a) amide, (b) boronate ester, (c) imine, and (d) imidazole linkages.

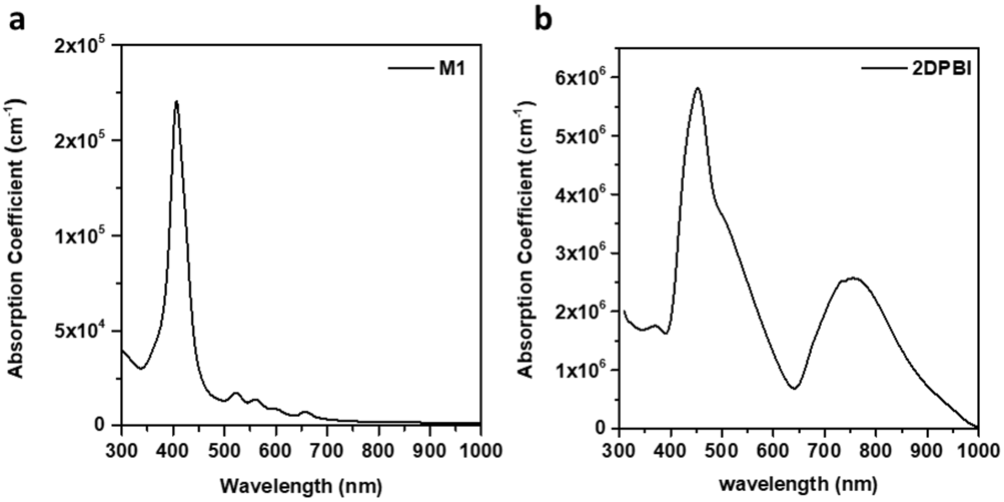

Figure S3. Absorption coefficient of (a) pre-assembled **M1** porphyrin monomer film (b) 2DPBI film.

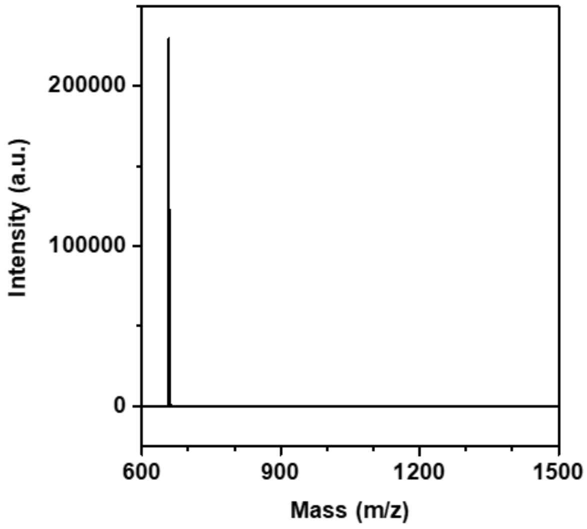

Figure S4. MALDI TOF MS analysis of the same model chemical reaction was performed in water under similar experimental conditions (pH, temperature, time, and concentration) without using a surfactant monolayer.

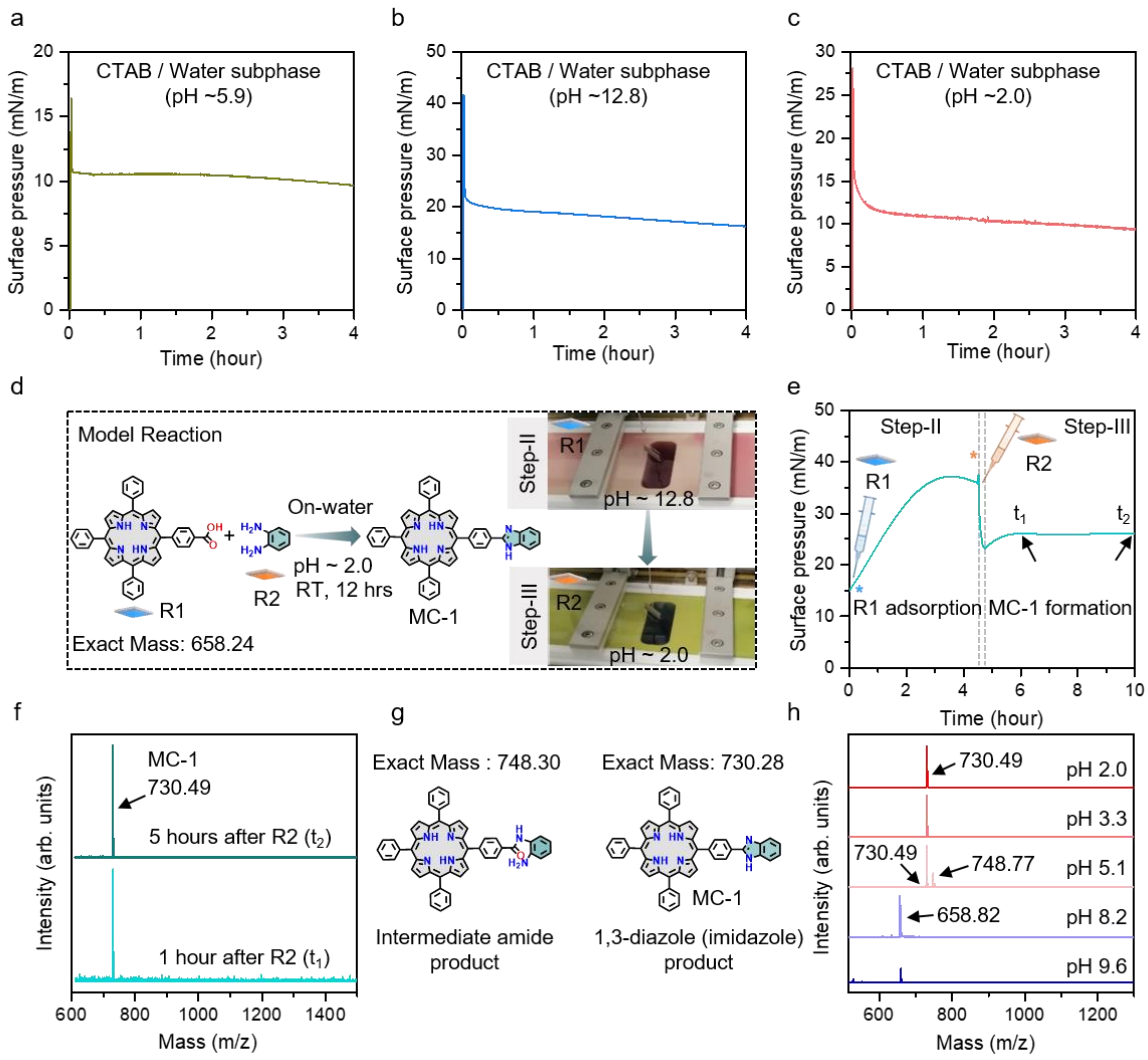

Figure S5. (a-c) Time-dependent surface pressure measurements of the CTAB surfactant monolayer on the water surface under different pH conditions. (d) Schematic illustration of the model reaction pathway depicting 1,3-diazole (imidazole) bond formation. (e) Surface pressure evolution during Step-II (injection of R1) and Step-III (injection of R2) on the water surface. (f) Time-dependent MALDI-TOF mass spectra of MC-1 collected after 1 hour and 5 hours, confirming 1,3-diazole formation on the water surface. (g) Molecular structures of the intermediate amide and the final 1,3-diazole products. (h) pH-dependent study of the 1,3-diazole reaction.

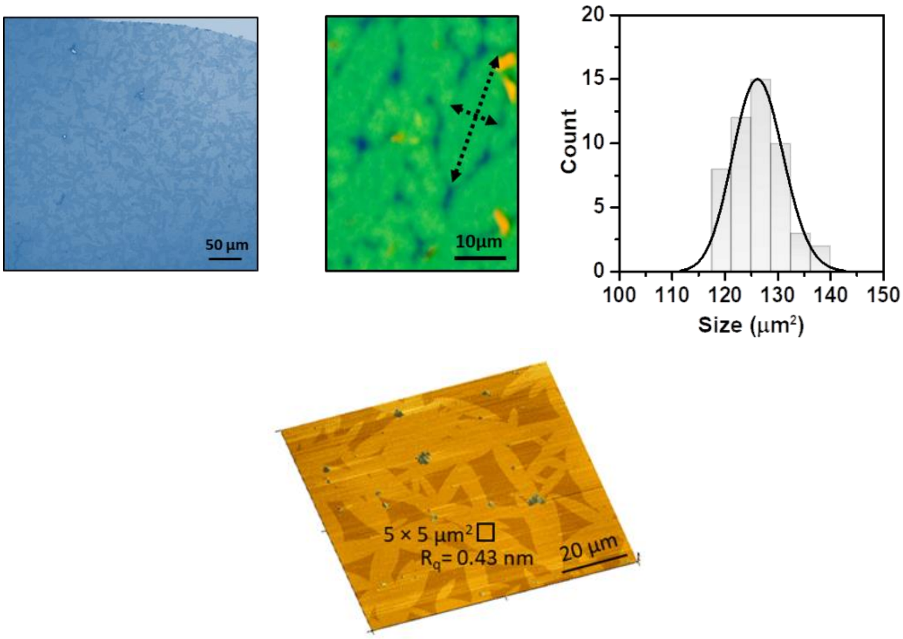

Figure S6. Optical microscope image of 2DPBI synthesized on the water surface with size distribution. AFM analysis, which revealed a uniform distribution of 2DPBI crystals with a surface roughness (Rq) of 0.43 nm measured over an area of 5 × 5 μm².

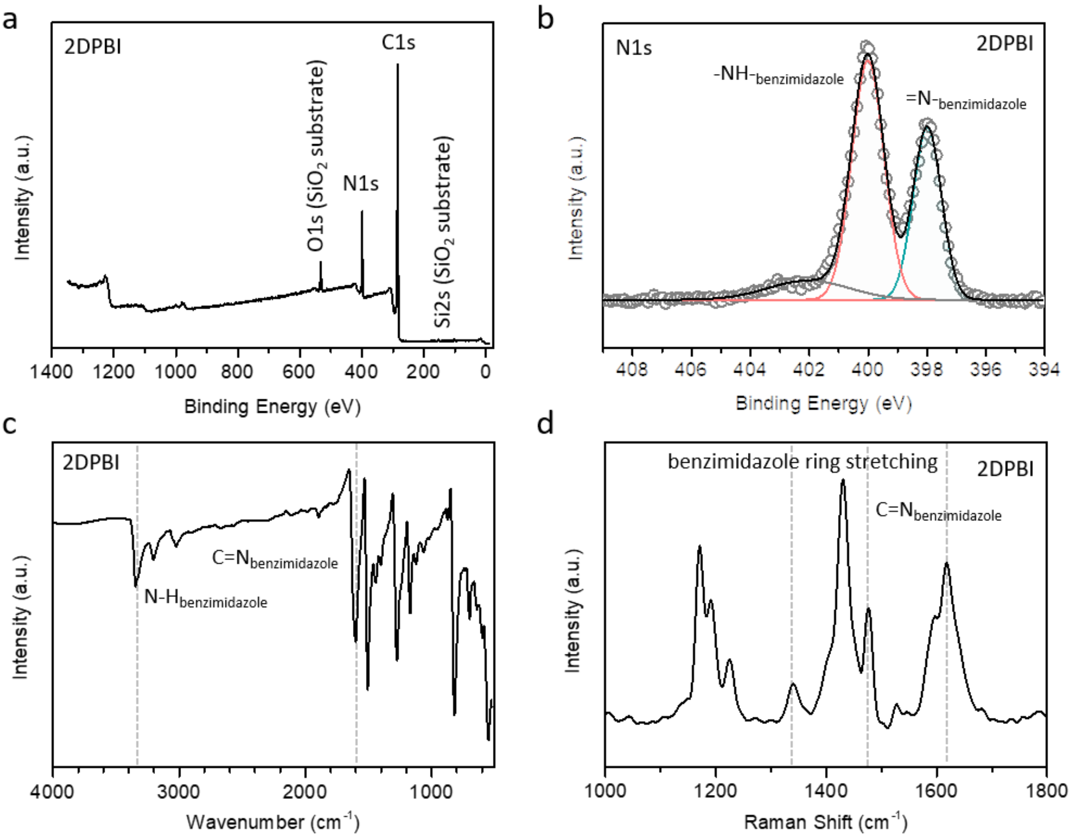

Figure S7. XPS analysis of 2DPBI thin films. (a) Full survey spectrum showing peaks for C1s, N1s, and the SiO_2 substrate. (b) High-resolution N1s spectrum displaying peaks at 400.2 eV and 398.1 eV, corresponding to the amine and imine nitrogen atoms within the 1,3-diazole linkage, respectively. (c) FTIR spectrum of the 2DPBI film. (d) Raman spectrum of the 2DPBI film.

The XPS spectra clearly showed peaks for C1s, N1s, and the substrate SiO_2, as the thin films are only 2.5 nm thick. The substrate signal is also evident in the full survey spectrum. In the high-resolution N1s spectrum, peaks at 400.2 and 398.1 eV were observed, corresponding to the amine and imine nitrogen atoms of the 1,3-diazole linkage, respectively. Further comparative analysis using FT-IR spectroscopy revealed a characteristic peak for the hydrogen-bonded N–H group within the 1,3-diazole (imidazole) ring. Additionally, pronounced new bands at 1628 cm^{-1} were attributable to the C═N vibrations in the 1,3-diazole ring structure. Similarly, Raman spectroscopy identified prominent bands between 1400 and 1650 cm^{-1} (γ region), with the most intense peaks at 1480 and 1617 cm^{-1}. These bands were assigned to benzimidazole ring stretching vibrations, as well as C=C and C=N stretching modes.

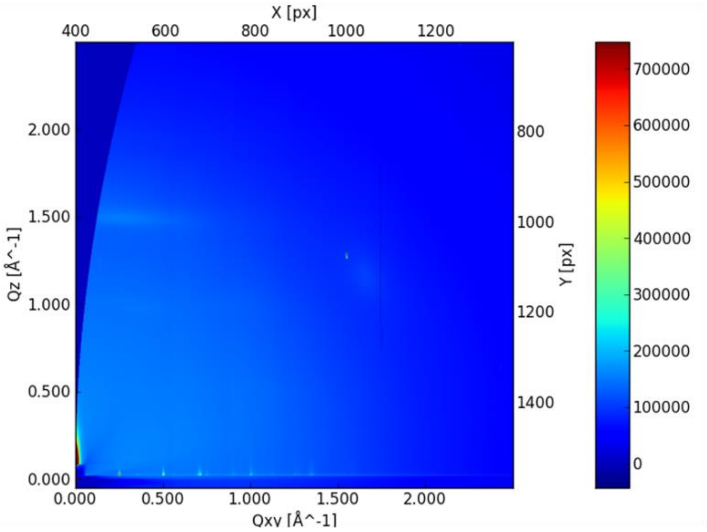

hk0	Q_{hk0} [A^{-1}]	d_{hk0} [A]
01	0.252	24.93
11	0.356	17.65
02	0.503	12.49
21	0.562	11.18
22	0.710	8.85
03	0.751	8.37
23	0.902	6.97
14	1.030	6.10
42	1.122	5.60
34	1.252	5.02
15	1.278	4.92
25	1.348	4.66
06	1.500	4.19
16	1.523	4.13

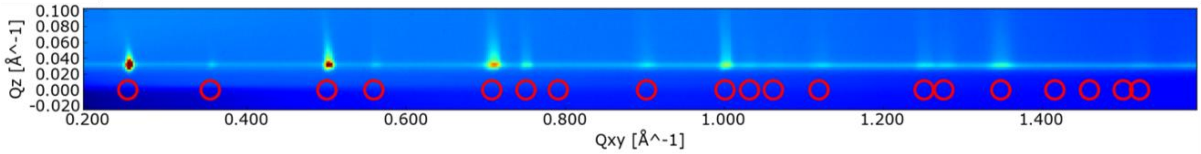

Figure S8. The corresponding measured GIXD scattering profile of 2DPBI film exhibited twelve distinct and sharp diffraction peaks along with dxy [Å] values.

Stacking in Layered 2DPBI

We started the stacking analyses with the DFTB method to speed up the calculations and used the geometries obtained in the DFT calculations. The results obtained with the two methods are consistent with each other. Here, we will only discuss DFT results for stacking analyses.

In order to determine the type of stacking in layered-bulk polybenzimidazole (PBI) COF, we compared the ground state energies of three different stacking types: eclipsed AA-stacking, inclined AA-stacking, and staggered AB-stacking. The structures are shown in **Supplementary Fig. 7 (a)-(c)**. The difference between the ground state energies of different stacking types is given in **Supplementary Fig. 8 (a)**. The inclined AA-stacking is the most favorable stacking type among these three since it has the lowest ground state energy.

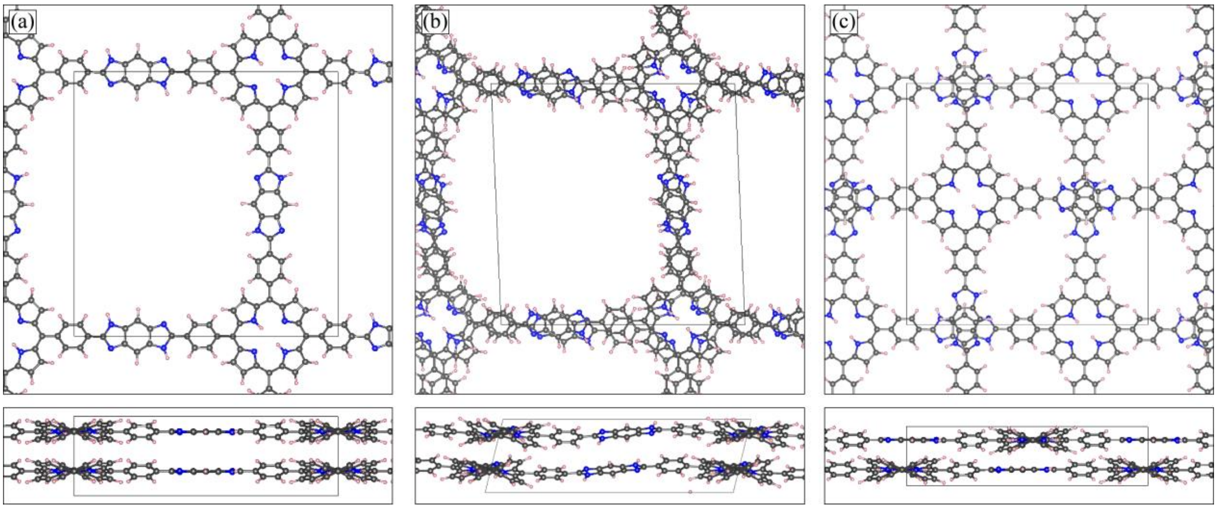

Figure S9. Different types of stacking in polybenzimidazole (PBI) COF: (a) eclipsed AA-stacking, (b) inclined AA-stacking, and (c) staggered AB-stacking. Dark grey, blue, and pink atoms represent C, N, and H atoms, respectively.

The obtained lattice parameters of AA_{inc} 2DPBI are a = b = 2.49 nm, c = 0.77 nm, α= 77, β= 79, and γ=90°. In AA_{inc} 2DPBI, the distance between porphyrins in two adjacent layers is 3.88Å, and the shortest distance between layers in two adjacent layers is 1.05Å. The calculated pore size is 2.55 nm.

AA_{inc} 2DPBI has a direct band gap at *M* high symmetry point. The band gap value calculated with PBE is 1.07 eV. As seen in Figure S9 (c), conduction and valance bands are mainly composed of C-*pz*. Partial density of states (PDOS) calculations reveal the hybridization between C-*pz*, C-*px*-*py*, and N-*pz* states in conduction and valance bands. The splitting in conduction bands is 13.8 meV, and 50.3 meV in valence bands.

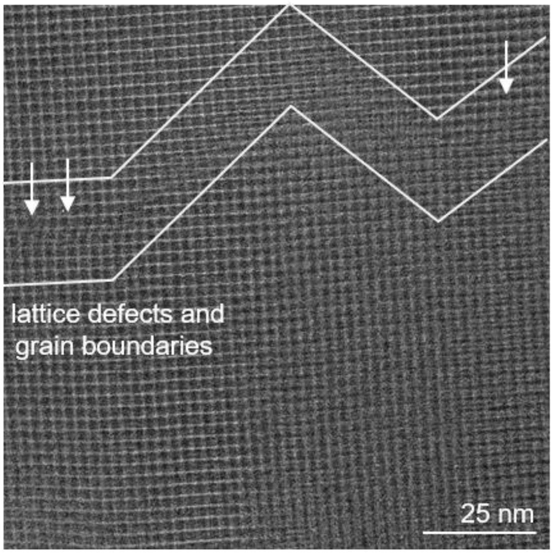

Figure S10. Lattice defects and grain boundaries were observed in overlapping regions of the crystals.

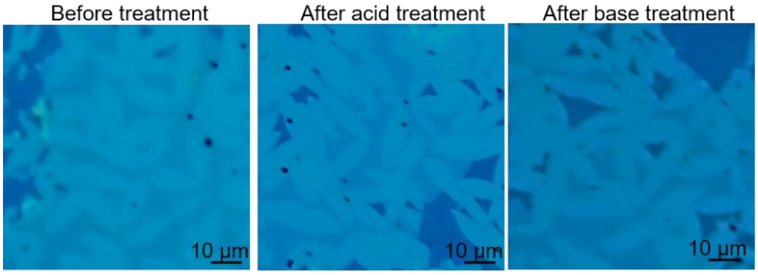

Figure S11. Optical microscopy images of 2DPBI thin film (a) before treatment, (b) after immersion in a strong acidic aqueous solution (12 M HCl), and (c) after immersion in a strong basic aqueous solution (12 M NaOH) for 1 day. The morphology remains well preserved, indicating the high chemical stability of 2DPBI due to its irreversible imidazole linkages.

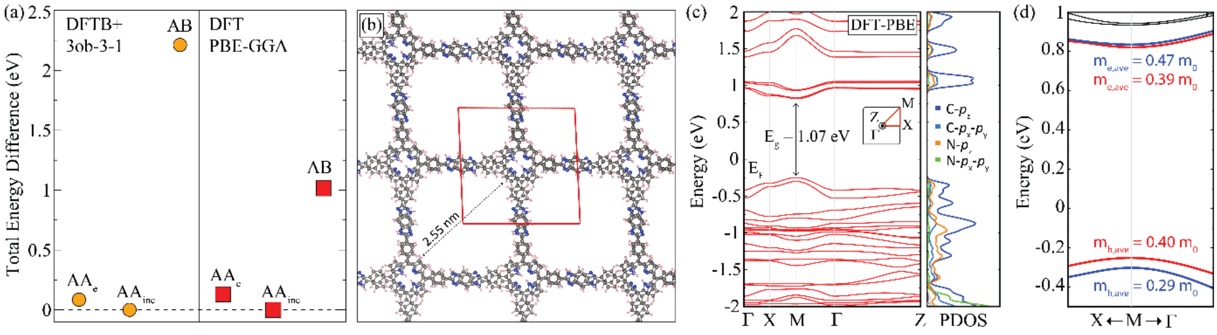

Figure S12. (a) Total energy difference for different stacking types: eclipsed AA-stacking (AA_e), inclined AA-stacking (AA_{inc}) and staggered AB-stacking (AB). Total energy of AA_{inc} is set to zero. (b) The top-view (upper) and side-view (under) of AA_{inc} PBI. (c) The calculated electronic band structure and partial density of states (PDOS) in arbitrary units are shown. Fermi level E_F is set to zero. Brillouin zone and the selected k-path is shown as an inset figure. (d) The effective mass values for conduction and valence bands are 0.39 and 0.40 m_0, respectively.

Average effective mass values, m^*_{avg} , were calculated via geometric mean, $m^*_{avg}=(m_{M\text{-}X}\, m_{M\text{-}\Gamma})^{1/2}$ where $m_{M\text{-}X}$ and $m_{M\text{-}\Gamma}$ are the effective mass values along *M-X* and *M-Γ* directions. The calculated average effective mass values for electrons and holes are 0.39 and 0.40 m_0, respectively.

Vibrational and Mechanical Properties of Monolayer 2DPBI

The density functional perturbation theory (DFPT) is commonly used to analyze phonon modes. However, this method can be computationally expensive for materials that have a large number of atoms in their unit cells, such as the monolayer 2DPBI, which has 106 atoms in its unit cell. Here, we used the DFTB method along with the small displacement method to calculate force constants. This approach is more computationally efficient and allows us to analyze phonon modes in large and complex materials.

The vibrational analyses showed that the monolayer 2DPBI is dynamically stable. In 2D materials, 3N-3 optical phonon branches are in their spectrum, where N is the number of atoms in the unit cell. All phonon branches are shown in Figure S13 (a). For monolayer 2DPBI, there are three acoustic and 315 optical phonon branches. The maximum phonon frequency is 421 meV. There are 32 optical phonon branches up to 25 meV (see Figure S13 (b)). There is no gap between acoustic and optical phonon branches, and they overlap between 0.7 and 3.5 meV.

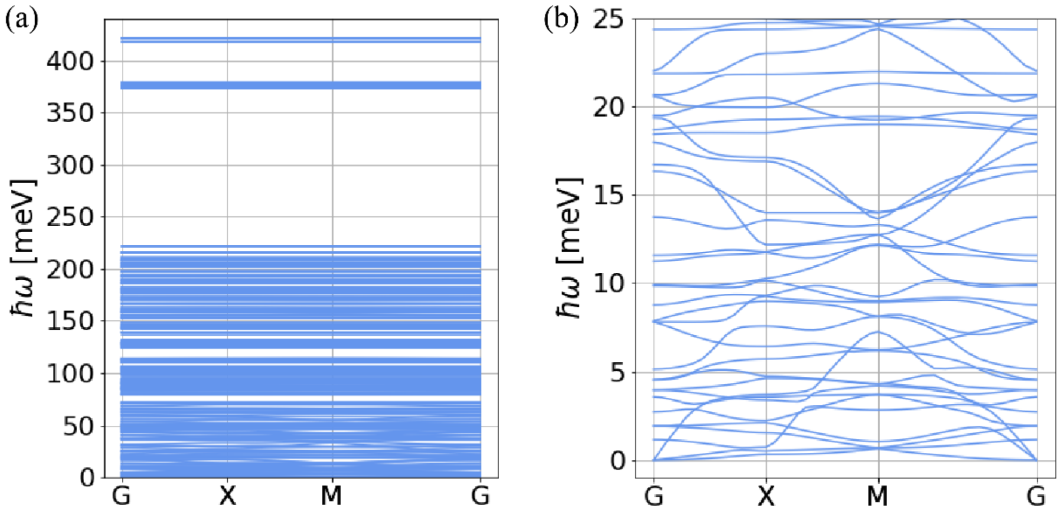

Figure S13. Phonon band structure for 3x3x1 supercell: the phonon branches (a) up to 440 meV and (b) up to 25 meV.

In order to investigate the in-plane stiffness of the material, we calculated 2D Young's modulus Y_{2D} with linear compression. Using quadratic regression, the data set was fitted to the elastic energy formula within the harmonic region[3]. The structure was then subjected to 1% of uniaxial and biaxial tension and compression.

When the material is assumed to be isotropic, Y_{2D} calculated with DFT (DFTB) is approximately 23 (26) N/m.

Nonparabolic Electronic Bands of Monolayer 2DPBI

It is generally assumed that the bands are parabolic around the band edges. However, nonparabolicity can significantly affect the transport properties of materials[4,5]. One method of analyzing the nonparabolicity is the Kane band model[6]. Here, we used effmass code[7] in order to investigate the nonparabolicity in electronic bands.

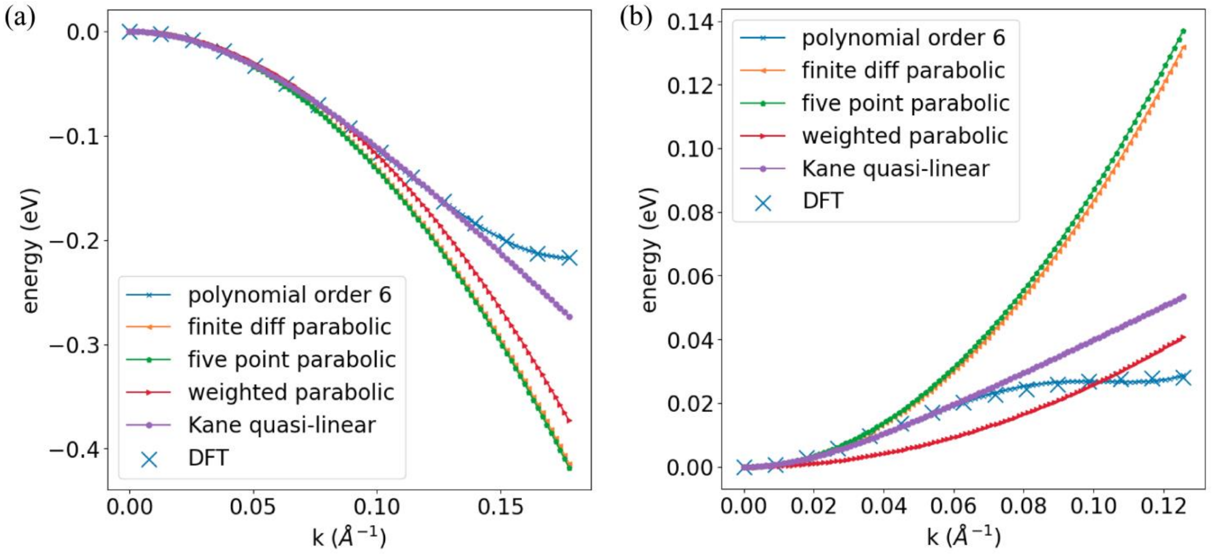

Figure S14. (a) The valence and (b) the conduction band dispersion calculated with DFT for the monolayer PBI.

As seen in Figure S14, the 6th-order polynomial model provides the best fit to the DFT results, but using it to reconstruct the transport properties with this model can be quite challenging. The Kane quasi-linear approximation is valid until -0.14 eV for the valence band (see Figure S14 (a)), and 0.02 eV for the conduction band (see Figure S14 (b)). The parabolic approximation is applicable up to approximately -0.09 eV for the valence band. In the case of the conduction band, its validity is limited to a region proximity to the band edge.

The effective masses calculated with 3-point finite difference method are 0.29 and 0.46 m_0 for the holes (m_h) and the electrons (m_e), respectively. These results are consistent with the results obtained with DFT (m_h=0.29 < m_e=0.42) and DFTB (m_h=0.47 < m_e=0.85) within the parabolic band assumption. Assuming the Kane bands, the optical mass at the band edge is 0.32 (0.79) m_0 for the holes (electrons). The Kane mass at the band edge is 0.28 (0.46) m_0 for the holes (electrons).

The nonparabolicity constant α determined via DFT and DFTB methods are 2.18 1/eV and 5.5 1/eV, respectively. Parameter α obtained using DFTB is larger, as the DFTB method computes band structures with a slightly widened profile.

Transport Properties of Monolayer 2DPBI

Here, we present the results on transport properties calculated by solving the Boltzmann equation within the constant relaxation time approximation (CRTA) as implemented in DFTBephy package[8].

For the fixed density, we perform data interpolation. Charge carrier mobility is a function depending on potential μ and temperature T. With density being fixed, we determine the chemical potential from the density (which also depends on μ and T). Subsequently, we can compute the mobility as a function of μ and T for the determined chemical potential. The densities as a function of interpolated chemical potentials at different temperatures are shown in Figure S15. Then, we calculated the densities and the conductivities of the charge carriers by assuming a constant relaxation time with two sets of chemical potentials: fixed chemical potentials and interpolated chemical potentials for fixed densities. Densities calculated for different temperatures with fixed chemical potential and chemical potentials obtained by interpolation are given in Figure S16.

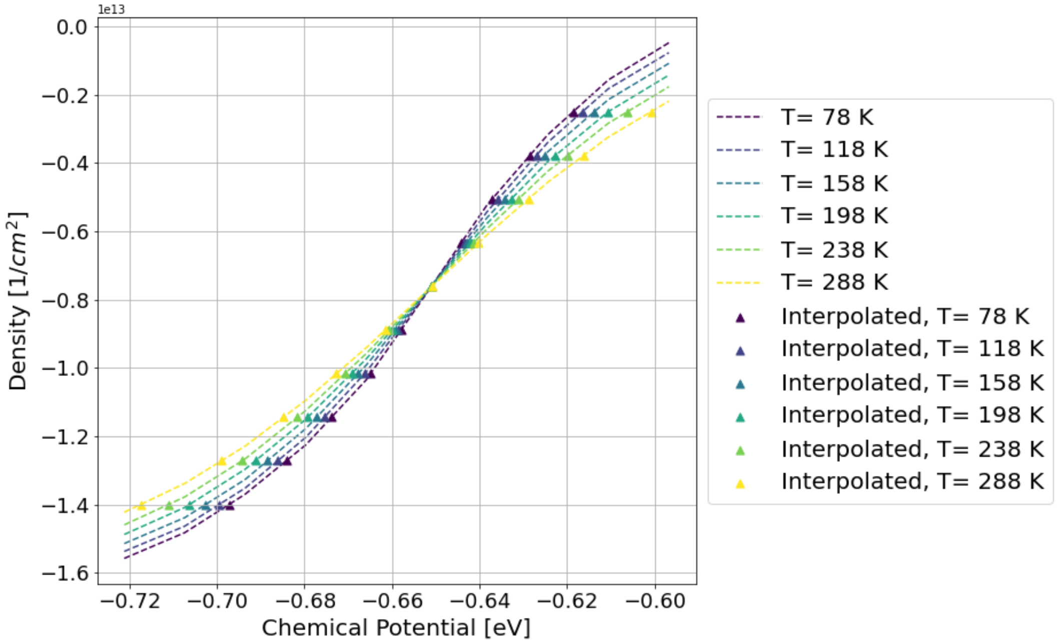

Figure S15. Interpolated chemical potentials vs densities at different temperatures for holes.

At a constant temperature, when the chemical potential moves into the band, the density is expected to increase. When the chemical potential moves away from the band, below the lower band edge for holes, the density is projected to remain constant. Additionally, when the chemical potential is fixed near the top of the valence band, an increase in density is expected as the temperature rises. However, near the lower band edge, the density may remain stagnant or even decrease due to the depopulation of hole states within the band at high temperatures. This behavior is not symmetric since the valence band dispersion is different at the two edges.

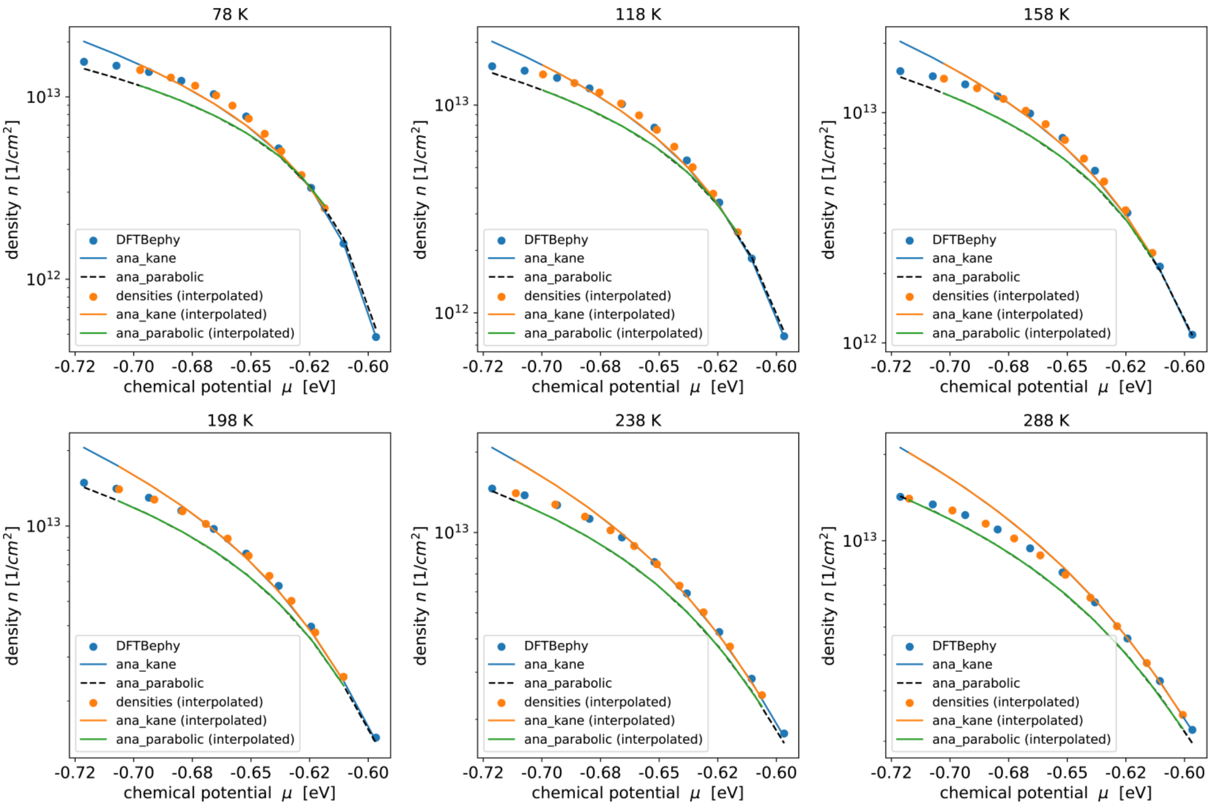

Figure S16. Densities as a function of chemical potential at different temperatures for holes. The nonparabolicity constant α is 5.5 1/eV.

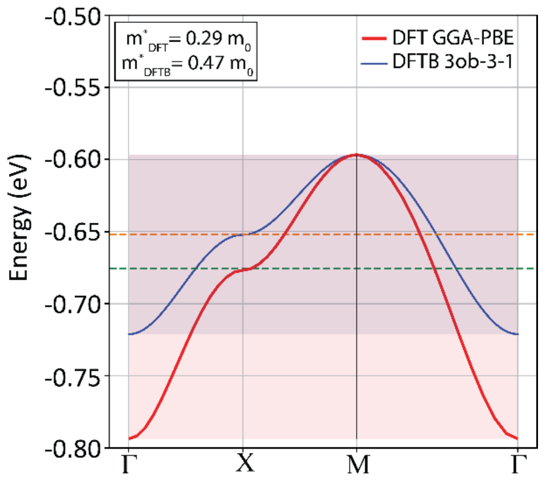

Figure S17. The valence band dispersion calculated with the DFT GGA-PBE functional and DFTB 3OB parametrization set. The effective mass values of holes m^* calculated with the DFT and DFTB methods are 0.29 and 0.47 m_0, respectively.

The analysis concerning conductivity follows a similar framework. In a fully occupied band, conductivity is zero due to the cancellation of currents from holes or electrons moving in opposite directions. At the midpoint of the band, conductivity reaches its maximum value. Consequently, when the chemical potential remains fixed near the band center, a decrease in

conductivity is expected, whereas it increases near the band edges as temperature increases. The behavior of mobility follows from the aforementioned analysis. In conductivity and mobility calculations we used the experimental value for the constant relaxation time, which is 61 fs (at 288 K).

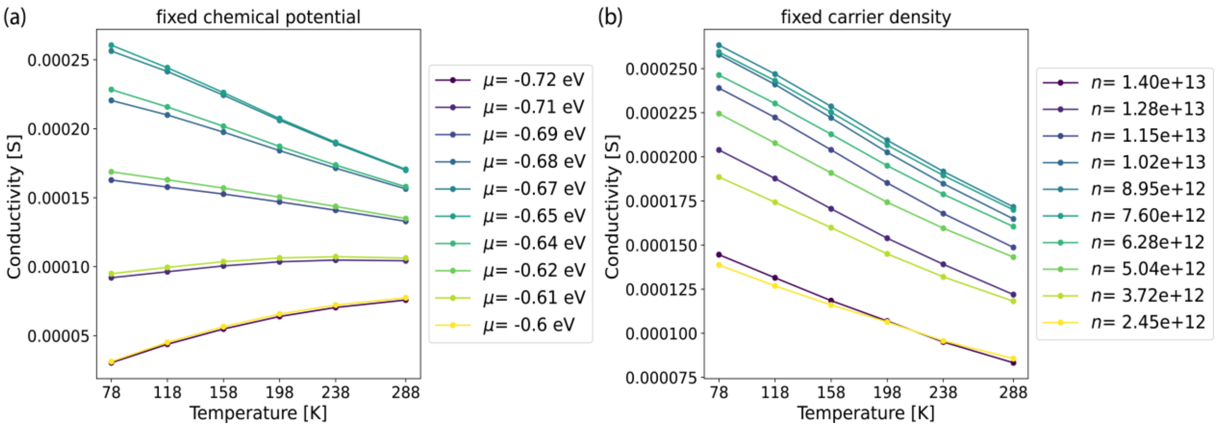

Figure S18. Conductivities as a function of temperature for holes: (a) with fixed chemical potentials, and (b) with fixed carrier densities. We used the experimental value for the constant relaxation time at 288 K, 61 fs.

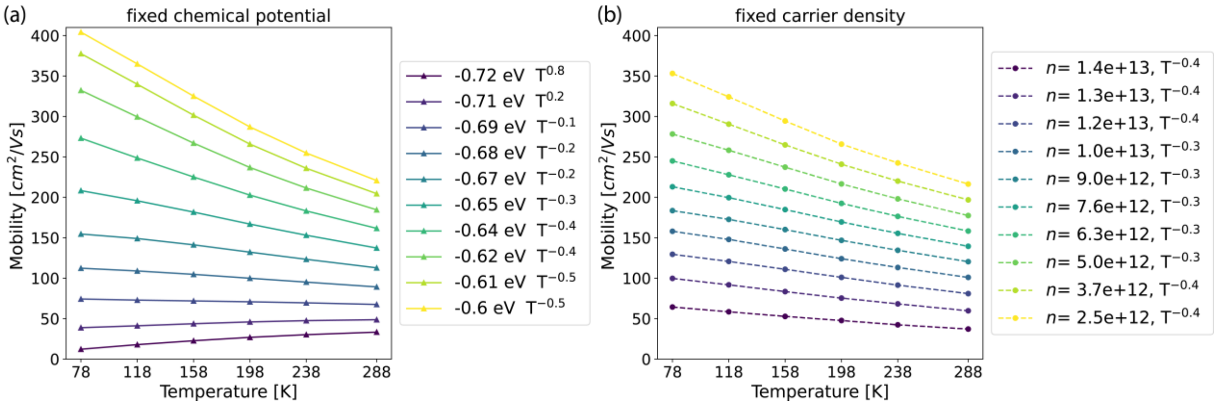

Figure S19. Mobilities as a function of temperature for holes: (a) with fixed chemical potentials, and (b) with fixed carrier densities, calculated with DFTBephy. We used the experimental value for the constant relaxation time at 288 K, 61 fs.

With the DFT method using the Boltztrap2 code[6], we can calculate the charge carrier mobilities only for the case where we keep the chemical potentials constant. In Figure S19 (a) we used the relaxation time value at 288 K. In Figure S19 (b), we used experimental relaxation times obtained for different temperatures. With the chemical potential at the valence band edge, the room-temperature mobilities of holes calculated via DFT and DFTB methods are 246 $cm^2V^{-1}s^{-}$

[1] and 220 cm^{2}V^{-1}s^{-1}, respectively. These values are in accordance with the experimental measurement of 240 cm^{2}V^{-1}s^{-1}.

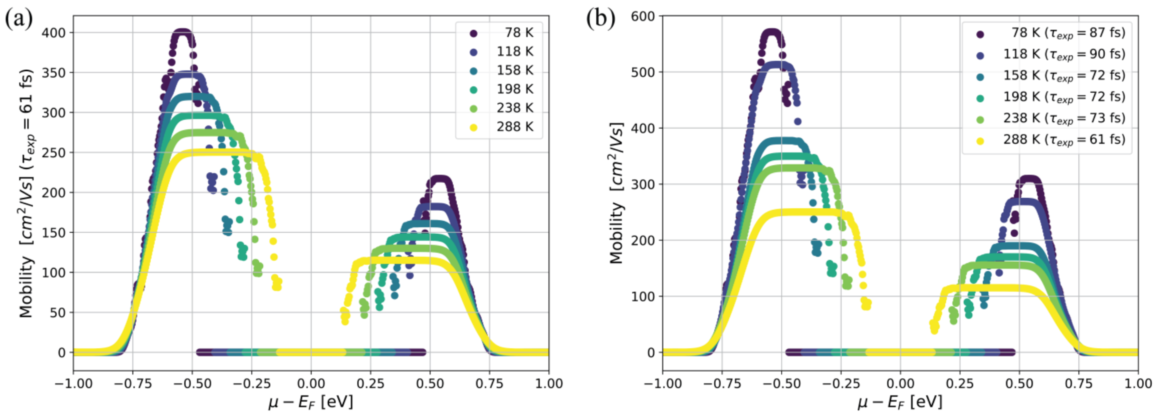

Figure S20. Mobilities calculated with DFT as a function of temperature for holes: (a) with a constant relaxation time of 61 fs, and (b) with the experimental values of the temperature dependent relaxation times. Conductivities were calculated with BoltzTraP2 code[9].

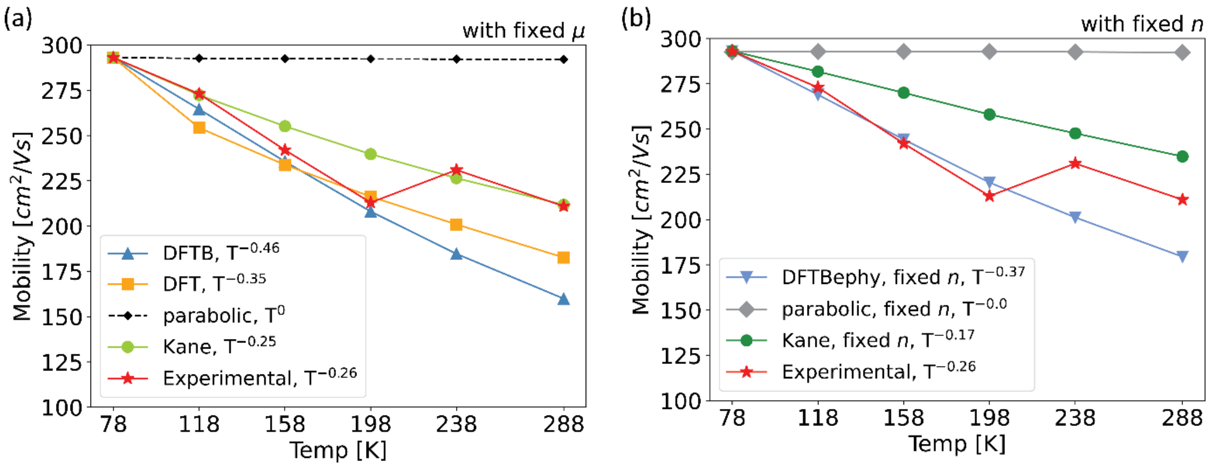

Figure S21. Room-temperature mobilities calculated with DFT as a function of temperature for holes: (a) with fixed chemical potential μ and (b) with fixed density n. The fixed densities at different temperatures.

Temperature-dependent mobilities were then calculated using Boltzmann transport theory under the constant relaxation time approximation (CRTA). Our theoretical results reveal that the temperature dependence of mobility is notably influenced by the presence of non-parabolicity. Mobilities with the Kane-band model exhibit a temperature dependence of $T^{-0.25}$ when the chemical potentials are fixed. With a set of fixed carrier densities, the mobility demonstrates a proportionality to $T^{-0.17}$. This difference arises from an additional effective temperature dependence of the chemical potential for the fixed densities (doping levels). As the

temperature rises, the energy window of the chemical potential widens. Moreover, the temperature decay increases with increasing non-parabolicity. Our calculations affirm that mobility estimations using the CRTA align consistently with experimental observations.

The charge mobility increases as the temperature decreases from 288 to 78 K. This is a clear indication of the band-like transport of charge carriers in 2DPBI. For band transport, the charges are largely delocalized and scattered mainly by phonons at finite temperatures. With decreasing temperature, the phonons are gradually frozen and phonon scattering is suppressed, so the charge mobility increases. In contrast, in the hopping transport mechanism, the charges are strongly localized at unit cells and can only hop from one cell to another. By increasing the temperature, the thermal excitation can enhance the hopping rate, so the charge mobility increases with rising the temperature. This result provides a strong evidence of charge delocalization and strong molecular conjugation in 2DPBI.

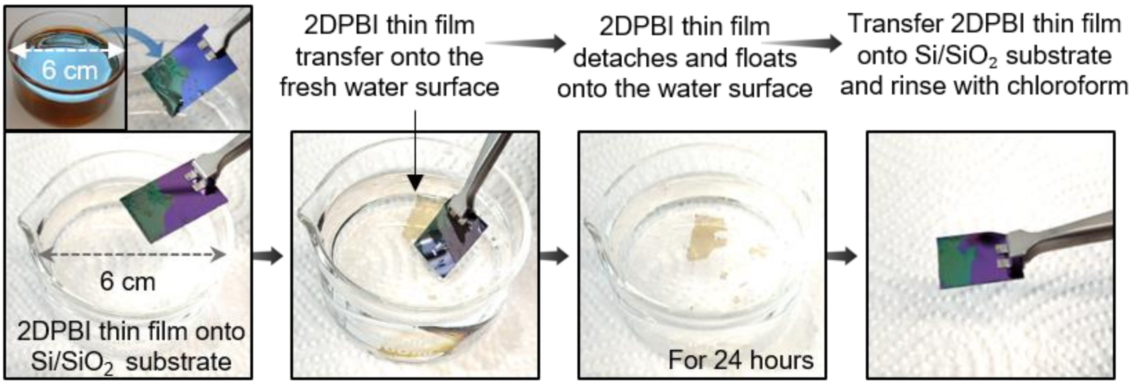

Figure S22. Sequential images showing the cleaning process of the 2DPBI thin film.

The synthesized 2DPBI thin film was initially transferred from the water surface to a Si/SiO_2 substrate using the Langmuir-Blodgett (LB) or Langmuir-Schaefer (LS) method. Without allowing the wet-transferred film to dry on the substrate, the substrate was gently brought back into contact with a fresh Milli-Q water subphase, enabling the 2DPBI film to detach and float back onto the clean water surface. The 2DPBI thin film was then left on the fresh water subphase for 24 hours to remove physisorbed and unreacted monomers. After this cleaning step, the 2DPBI film was transferred onto a Si/SiO_2 (300 nm) substrate. Following the final transfer, the film was carefully rinsed with chloroform to eliminate any remaining unreacted monomers or surfactants. Subsequently, 50 nm thick Au contacts were thermally evaporated onto the 2DPBI films under high vacuum conditions (~10^{-7} mbar) at a deposition rate of 1.5 Å s^{-1}, with the substrate temperature maintained at room temperature.

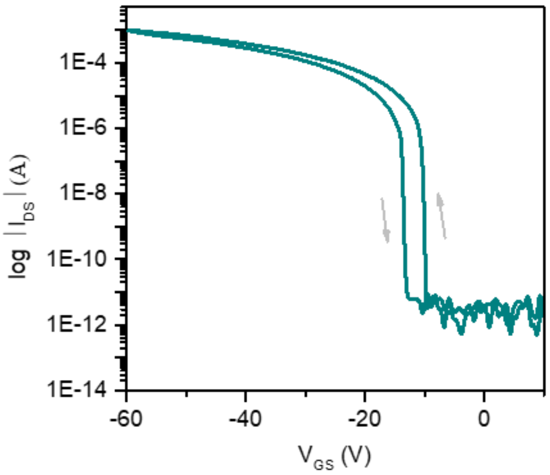

Figure S23. Current versus gate voltage sweep curves at an applied drain voltage of -60V for the channel (20 µm) 2DPBI transistors.

Optical figures of merit:

The photosensitivity, P, and photoresponsivity, R, are expressed as in equation 1 & 2,

$$P = \frac{signal}{noise} = \frac{Id,illumination - Id,dark}{Id,dark} \qquad 1$$

$$R = \frac{Iph}{Pinc.A} = \frac{Id,illumination - Id,dark}{Pinc.A} \qquad 2$$

where $I_{d,illumination}$, $I_{d,dark}$, P_{inc} and A are the current measured under illumination, in dark, the incident light intensity, and area of the active region, respectively.

The specific detectivity, D*, is calculated from equation 3 as,

$$D^* = \frac{(R\sqrt{A})}{\sqrt{2qId,dark}} \qquad 3$$

Where q is the charge of the electron. The calculations were done considering that the overall background noise is dominated by the dark current.

Using the above equations 1, 2 and 3; P, R and D* have been calculated and plotted with respect to the applied voltage.

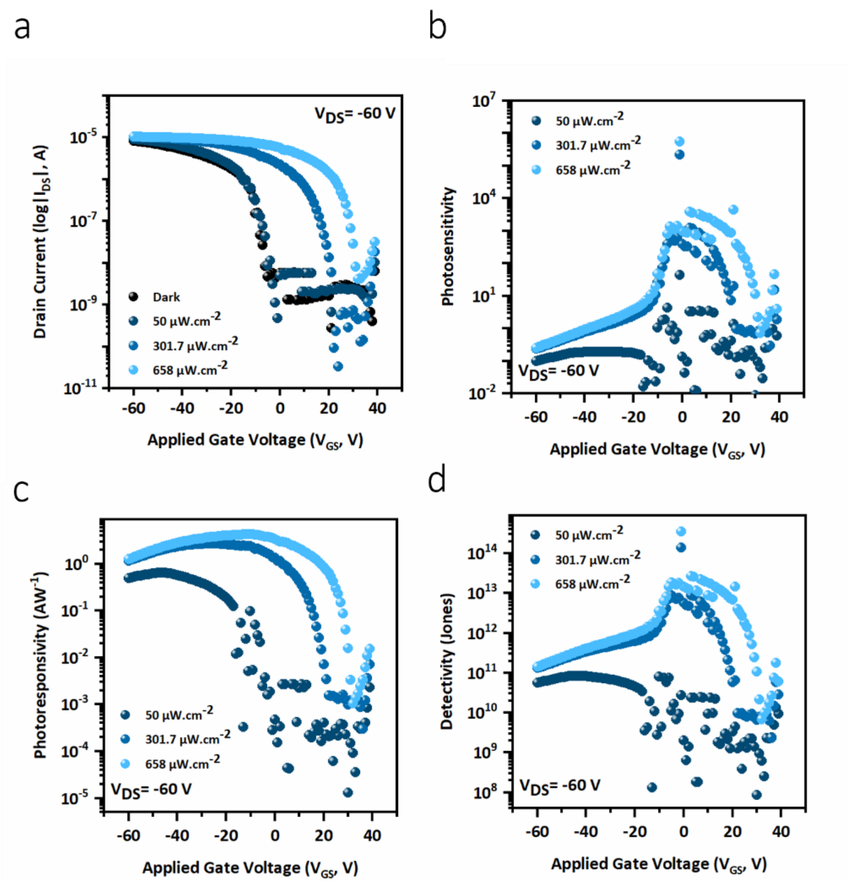

Figure S24. The a) transfer characteristics of 2DPBI films (long channel device, 300 µm) under 455nm blue light at different illumination intensities and the b) photosensitivity, c) photoresponsivity and d) detectivity derived from a).

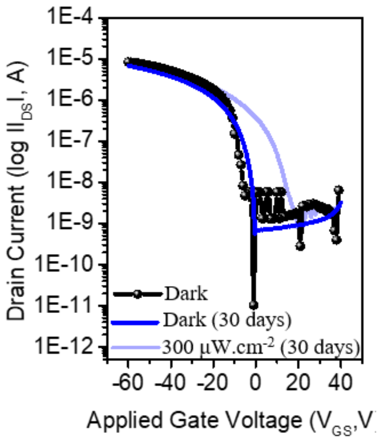

Figure S25. Long-term stability of FET and phototransistor devices based on 2DPBI. The transfer characteristics (drain current vs. gate voltage) show stable performance after 30 days.

The long-term stability of the FET and phototransistor devices based on 2DPBI, as shown in Supplementary Fig. 20, which demonstrate stable device performance for both the FET and phototransistor over a period of one month. The sample was stored for a month under controlled environmental conditions with a temperature of 20 ± 2 °C and humidity of 40 ± 10%.

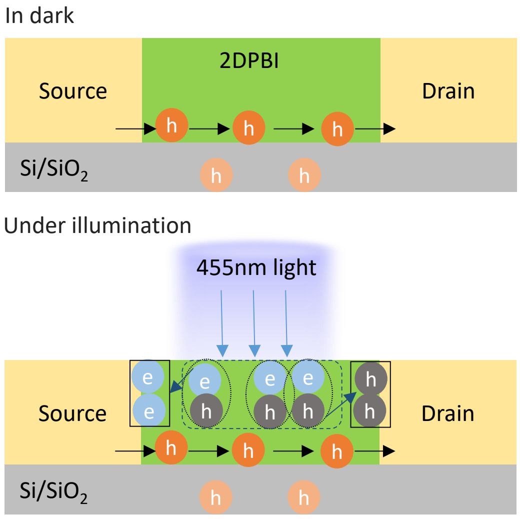

Figure S26. Schematic representation of the charge transport process under dark and illuminated conditions, highlighting hole transport in the dark and electron-hole pair generation under 455 nm illumination.

Chemical Design Enabling Photoresponse Mechanism

The rapid photoresponse in 2DPBI devices originates from the unique chemical design of the 2DPBI structure, which facilitates photogating via shallow trap states while preserving band-like charge transport essential for rapid photoconductive operation. 2DPBI is a conjugated

polybenzimidazole-based 2D polymer consisting of a π-conjugated porphyrin core covalently linked by 1,3-diazole (imidazole) bonds, reinforcing intrinsic charge delocalization and band-like transport characteristics. Strong donor-acceptor (D–A) interactions within this structure enable efficient exciton dissociation, critical for high-sensitivity photodetection. Specifically, the porphyrin units serve as electron donors, while the imidazole units act as electron acceptors. The fully conjugated structure enhances π-electron delocalization, resulting in a narrow bandgap (~1.18 eV), low electron-hole effective mass ($m^* = 0.171\ m_0$), and high intrinsic mobility (~240 $cm^2V^{-1}s^{-1}$). Consequently, photogenerated charge carriers in 2DPBI rapidly transport through the continuous energy bands in a delocalized manner. Simultaneously, the porphyrin backbone provides photoactive sites that temporarily trap photogenerated electrons, inducing a photogating effect (threshold voltage shift) under illumination. Crucially, the strategic chemical design and single-crystal thin-film quality (device channel length: 20 μm) minimize both the depth and density of trap states, resulting in shallow traps that enhance device sensitivity through effective channel gating, while strong light absorption (up to 10^6 cm^{-1}) significantly increases the device's light–matter interaction capability.

Shallow Traps and Band-Like Transport for Fast Response

The shallow trap states in 2DPBI, located close to the band edge, enable easy release of trapped photogenerated electrons once illumination is removed. Consequently, the photogating-induced charges dissipate within milliseconds, ensuring the threshold voltage shift reverses almost immediately after turning off the light, without leaving significant long-lived charges in the device. Simultaneously, the band-like transport characteristics of 2DPBI ensure that mobile carriers (holes in the p-type channel) propagate quickly under the action of the electric field. Such effective charge delocalization prevents strong carrier-trap interactions during device operation, facilitating rapid photocurrent rise and decay, even in the presence of photogating. In transient measurements, notably, the decay curve rapidly returns to the dark baseline without an extended tail, indicating rapid recombination of photogenerated carriers once illumination ceases. The clean, mono-exponential decay observed in 2DPBI devices occurs on the millisecond scale, confirming that photogating traps do not retain charge after illumination is removed. We also carried out repetitive on/off pulsing experiments (Supplementary Fig. 25), which show perfectly reversible switching with no cumulative build-up or memory effects. Each light pulse induces a similar current increase and the current fully decays before the next pulse. This reversible, quick recovery further confirms that any trapped charge in 2DPBI is short-lived. It is also important to note that the magnitude of the photogating shift can depend on device geometry and operating conditions, which helps reconcile the fast transients with the ~40 V Vth shift. The large shift in Supplementary Fig. 20 was measured in a long-channel device (L = 300 μm) under continuous illumination, where a greater number of traps can accumulate charge, enhancing the photogating effect in steady-state. In contrast,

our response time measurement was performed on a shorter-channel device, where the influence of trap capacitance is reduced and the intrinsic photoconductive response is more pronounced. Moreover, the response time was measured at $V_{GS} = 0$ (with a fixed source–drain bias), a condition under which the channel is initially off and the influence of trapped charge on the dynamics is reduced, allowing the fast band-like transport of photocarriers to dominate.

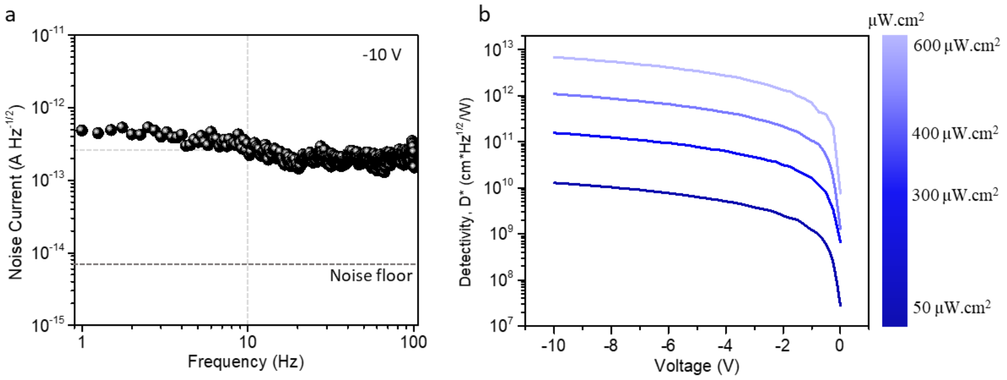

Figure S27. (a) Noise current as a function of frequency for the device with a short channel length of 20 μm. (b) Detectivity for different illumination intensities of the light source.

From the noise spectral density of the device (with a short channel length of 20 μm) and recalculated the specific detectivity based on Equation 1. At -10 V, the specific detectivity was determined to be 7.1×10^{12}.

$$D^* = \frac{\sqrt{A\Delta f}}{NEP} = R\frac{\sqrt{A\Delta f}}{I_{noise}}$$

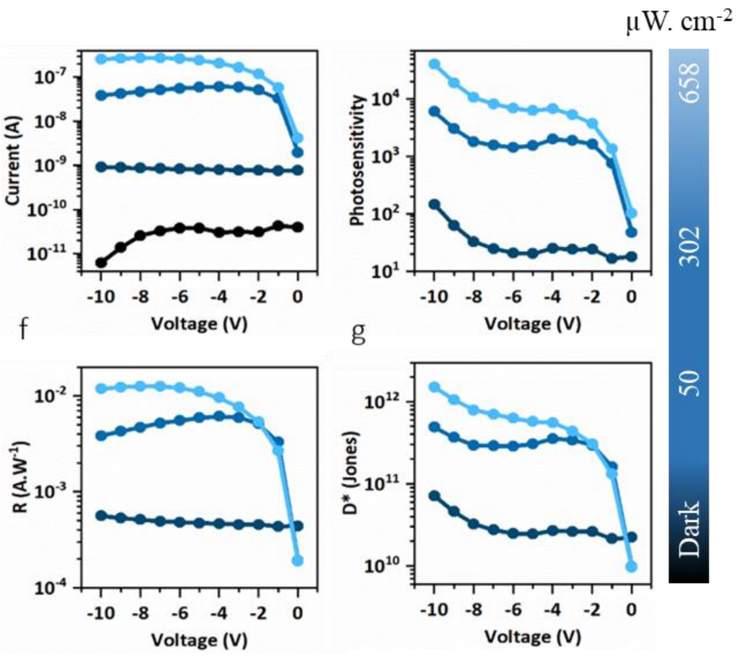

Figure S28. Electrical transfer characteristics for different illumination intensities of a long channel 2DPBI transistor. The channel length and width were 300 µm and 11000 µm, respectively. Current-voltage characteristics of 2DPBI layer at V_{GS}=0V indicating photodetection using a 455 nm light source followed by the calculated photosensitivity, photoresponsivity and detectivity for different illumination intensities of the light source. The measured photosensitivity and photoresponsivity values at V_{GS} = 0 V and V_{DS} = -10 V.

For a 300-µm channel length:

- The specific detectivity based on dark current is calculated to be 1.5×10^{12} Jones.

For a 20-µm channel length:

- The specific detectivity based on dark current is calculated to be 2.0×10^{13}Jones.
- The specific detectivity based on noise current is calculated to be 7.1×10^{12} Jones.

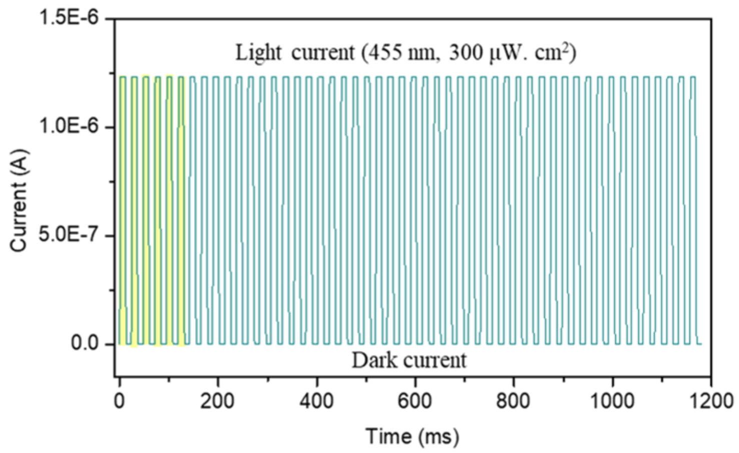

Figure S29. Photocurrent response under repeated illumination cycles. Transient response characteristics of the 2DPBI films with (shaded region) and without 455 nm light illumination.

Category	Published Year (Refs.)	Active Materials	Detectivity [Jones]
Graphene	2015 (58)	Graphene/dye R6G	1.5×10^{7}
	2017 (59)	Graphene/$Zn_{0.9}Mg_{0.1}O$ NPs/BET dye	1.7×10^{12}
	2017 (72)	Graphene/$Zn_{0.9}Mg_{0.1}O$ NPs/N_4 dye	1.3×10^{12}
	2017 (73)	Graphene/rubrene	9×10^{11}
	2018 (74)	Graphene/DPA	10^{13}
	2020 (75)	Graphene/C12 TBP	1.9×10^{9}
	2021 (76)	Graphene/HAT-CN/Bi2O2Se	1.2×10^{11}
	2022 (77)	Graphene/pentacene	$>10^{11}$
	2022 (78)	Graphene/BUBD-1	4×10^{11}
	2023 (79)	Rubrene/graphene	$>10^{12}$
	2016 (80)	Graphene/C8-BTBT	10^{8}
	2018 (81)	Graphene/PDA	6×10^{11}
	2020 (82)	Graphene/PTCDI-C8/Al	4.5×10^{9}
	2021(83)	Graphene/h-BN/J-aggregate PTCDI-C13	10^{10}
	2021 (84)	Graphene/h-BN/PTCDI-C13	10^{10}
	2021 (85)	Graphene/PDVF	10^{16}
	2023 (86)	PPy-NGr/SnO_2	6.47×10^{11}
	2015 (87)	Graphene/CH3NH3PbI3	10^{9}
	2018 (88)	Graphene/MOF	4.28×10^{13}
	2019 (89)	Graphene/P3HT:F4 TCNQ	1.31×10^{13}
	2020 (90)	Graphene/GNSs/MAPbI3	10^{13}
	2020 (91)	Graphene/TTF:CA	6×10^{13}
	2020 (92)	Graphene/COF	10^{13}
	2021 (93)	Graphene/Au array	2.43×10^{13}
	2021 (94)	Graphene/ZnO/IEICO-4F:PTB7-Th	10^{13}
	2022 (95)	Graphene/PM6:Y6	1.33×10^{12}
	2017 (96)	Graphene/PTCDA/pentacene	1.26×10^{9}
	2017 (97)	Graphene/C60/pentacene	1.8×10^{8}
MoS_2			
	2014 (98)	MoS_2/dye R6G	10^{10}
	2018 (99)	MoS_2/ZnPc	10^{11}
	2020 (100)	MoS_2/CuPc	2×10^{10}
	2022 (101)	DPA/MoS_2(DMDM)	3.5×10^{8}
	2022 (102)	MoS_2/2DPI	5.10×10^{12}
	2022 (103)	PbPc/MoS_2	1.4×10^{13}
	2023 (104)	MoS_2/BTP-4F	1.6×10^{9}
	2015 (105)	MoS_2/P(VDF-Tr18FE)	$\approx 2.2 \times 10^{12}$
	2017 (106)	MoS_2/g-C_3N_4	4×10^{11}
	2019 (107)	MoS_2/PDPP3T	3×10^{8}
	2023 (108)	PSS/SnS_2/MoS_2	6.31×10^{11}
2DPBI	This work	2DPBI (standalone 2D polymer)	7.1×10^{12} (Noise current based)
2DPBI	This work	2DPBI (standalone 2D polymer)	2.0×10^{13} (Dark current based)

Table S1 | Comprehensive comparison with high-performance optoelectronic materials.

Material	Bandgap	Mobility ($cm^2V^{-1}s^{-1}$)	Method	Refs.
2DCP-NiPC film	1.33	971 ± 44	THz	[10]
2DCP-CuPC film	1.28	460 ± 31	THz	
sp^2-COF	-	22.1 ± 2.7	THz	[11]
sp^2-COF-6	1.31	2.3 ± 0.5	THz	
sp^2-COF-8	1.33	<0.1	THz	
sp^2-COF-9	1.07	5.8 ± 0.9	THz	
I_2-sp^2-COF	-	51.1 ± 3.1	THz	
I_2-sp^2-COF-6	-	5.7 ± 0.3	THz	
c-HBC-COF	2.18	44	THz	[12]
DBOV-COF	1.74	0.6 ± 0.1	THz	[13]
V-2D-COF-W1	2.18	1.4	THz	[14]
V-2D-COF-W3	2.21	10.3	THz	
V-2D-COF-W4	2.26	0.6	THz	
I_2-V-2D-COF-W1	-	3.1	THz	
I_2-V-2D-COF-W4	-	1.2	THz	
PI-2DP 1	1.4	0.01	THz	[15]
TPB-TFB COF	2.6	165 ± 10	THz	[16]
HHTP-MIDA-COF	-	3.4	THz	[17]
CuPc-MIDA-COF	-	13.3	THz	
CuPc-pz COF	1.18	0.7 (h)	THz	[18]
ZuPc-pz COF	1.20	2.0 (h)	THz	
2DPAV-BDT-BT	1.62	~64.6	THz	[19]
2DPAV-BDT-BP	1.90	~16.8	THz	
CuPc-pz COF	1.18	0.9 ± 0.2 (h)	Hall	[20]
ZuPc-pz COF	1.20	4.8 ± 0.7 (h)	Hall	[20]
CuPc-MIDA-COF	-	8.2 (e)	Hall	[21]
COF-366	-	8.1 (h)	TRMC	[22]
COF-66	-	3.0 (h)	TRMC	
CS-COF	-	4.2 (h)	TRMC	[23]
H_2P-COF	-	3.5 (h)	TRMC	[24]
CuP-COF	-	0.19 (e)	TRMC	
ZnP-COF	-	0.032 (h)/0.016 (e)	TRMC	
NiPc COF	-	1.3 (h)	TRMC	[25]
HBC-COF	-	0.7 (h)	TRMC	[26]
TTF-Ph-COF	-	0.2 (h)	TRMC	[27]
TTF-Py-COF	-	0.08 (h)	TRMC	
2D D-A COF	-	0.01 (h)/0.04 (e)	TRMC	[28]
2DP (4)	1.4	1.3×10^{-6}	FET	[29]
I_2-2DP (4)	-	1.6×10^{-4}	FET	
2DPTTI	2.64	1.37×10^{-3}	FET	[30]
polyTB	~2.0	3.0×10^{-6}	FET	[130]
2DPBI	1.18	240 ± 38	THz	This work
2DPBI	1.18	0.17 ± 0.008	FET	This work

Table S2 | Comprehensive comparison of the selected semiconducting properties of reported 2DCPs.

2D COF and 2DP	Bandgap (eV)	Detectivity D* (Jones)	Refs.
$COF_{ETBC–TAPT}$-graphene	2.02	6.0×10^{13}	56
$TpDPP/TiO_2$	2.38	7.18×10^{9}	59
$TpEtBr/TiO_2$	2.26	7.94×10^{9}	59
$TpTab/TiO_2$	2.44	2.5×10^{7}	59
$TpTat/TiO_2$	2.50	4.4×10^{7}	59
2DPBI (standalone 2D polymer)	1.18	7.1×10^{12} (Noise current based)	This work
2DPBI (standalone 2D polymer)	1.18	2.0×10^{13} (Dark current based)	This work

Table S3 | Comprehensive comparison of high-performance optoelectronic materials, including 2D COFs and 2DPs, with calculations based on the same detectivity formula.

References

1. Hourahine, B. *et al.* DFTB+, a software package for efficient approximate density functional theory based atomistic simulations. *J. Chem. Phys.* **152** (2020).

2. Frenzel, J., Oliveira, A. F., Jardillier, N., Heine, T. & Seifert, G. Semi-relativistic, self-consistent charge Slater-Koster tables for density-functional based tight-binding (DFTB) for materials science simulations. *TU-Dresden* **2**, 7 (2009).

3. Topsakal, M., Cahangirov, S. & Ciraci, S. The response of mechanical and electronic properties of graphane to the elastic strain. *Appl. Phys. Lett.* **96** (2010).

4. Whalley, L. D., Frost, J. M., Morgan, B. J. & Walsh, A. Impact of nonparabolic electronic band structure on the optical and transport properties of photovoltaic materials. *Phys. Rev. B.* **99**, 085207 (2019).

5. Lundstrom, M. Fundamentals of carrier transport, 2000. *Cambridge, Cambridge Univ. press.* (2000).

6. Rudderham, C. & Maassen, J. Analysis of simple scattering models on the thermoelectric performance of analytical electron dispersions. *J. Appl. Phys.* **127** (2020).

7. Whalley, L. D. effmass: An effective mass package. *J. Open Source Softw.* **3**, 797 (2018).

8. Croy, A., Unsal, E., Biele, R. & Pecchia, A. DFTBephy: A DFTB-based approach for electron–phonon coupling calculations. *J. Comput. Electron.* **22**, 1231-1239 (2023).

9. Madsen, G. K. H., Carrete, J. & Verstraete, M. J. BoltzTraP2, a program for interpolating band structures and calculating semi-classical transport coefficients. *Comput. Phys. Commun.* **231**, 140-145 (2018).
